# Associations between Potentially Modifiable Risk Factors and Alzheimer Disease: A Mendelian Randomization Study

**DOI:** 10.1371/journal.pmed.1001841

**Published:** 2015-06-16

**Authors:** Søren D. Østergaard, Shubhabrata Mukherjee, Stephen J. Sharp, Petroula Proitsi, Luca A. Lotta, Felix Day, John R. B. Perry, Kevin L. Boehme, Stefan Walter, John S. Kauwe, Laura E. Gibbons, Alzheimer’s Disease Genetics Consortium, The GERAD1 Consortium, EPIC-InterAct Consortium, Eric B. Larson, John F. Powell, Claudia Langenberg, Paul K. Crane, Nicholas J. Wareham, Robert A. Scott

**Affiliations:** 1 Research Department P, Aarhus University Hospital, Risskov, Denmark; 2 Department of Clinical Medicine, Aarhus University, Aarhus, Denmark; 3 Department of Medicine, University of Washington, Seattle, Washington, United States of America; 4 MRC Epidemiology Unit, Institute of Metabolic Science, University of Cambridge, Cambridge, United Kingdom; 5 Department of Basic and Clinical Neuroscience, King’s College London, Institute of Psychiatry, Psychology and Neuroscience, London, United Kingdom; 6 Department of Biology, Brigham Young University, Provo, Utah, United States of America; 7 Department of Epidemiology and Biostatistics, School of Medicine, University of California San Francisco, San Francisco, California, United States of America; 8 Group Health Research Institute, Seattle, Washington, United States of America; Mount Sinai School of Medicine, UNITED STATES

## Abstract

**Background:**

Potentially modifiable risk factors including obesity, diabetes, hypertension, and smoking are associated with Alzheimer disease (AD) and represent promising targets for intervention. However, the causality of these associations is unclear. We sought to assess the causal nature of these associations using Mendelian randomization (MR).

**Methods and Findings:**

We used SNPs associated with each risk factor as instrumental variables in MR analyses. We considered type 2 diabetes (T2D, *N*_SNPs_ = 49), fasting glucose (*N*_SNPs_ = 36), insulin resistance (*N*_SNPs_ = 10), body mass index (BMI, *N*_SNPs_ = 32), total cholesterol (*N*_SNPs_ = 73), HDL-cholesterol (*N*_SNPs_ = 71), LDL-cholesterol (*N*_SNPs_ = 57), triglycerides (*N*_SNPs_ = 39), systolic blood pressure (SBP, *N*_SNPs_ = 24), smoking initiation (*N*_SNPs_ = 1), smoking quantity (*N*_SNPs_ = 3), university completion (*N*_SNPs_ = 2), and years of education (*N*_SNPs_ = 1). We calculated MR estimates of associations between each exposure and AD risk using an inverse-variance weighted approach, with summary statistics of SNP–AD associations from the International Genomics of Alzheimer’s Project, comprising a total of 17,008 individuals with AD and 37,154 cognitively normal elderly controls. We found that genetically predicted higher SBP was associated with lower AD risk (odds ratio [OR] per standard deviation [15.4 mm Hg] of SBP [95% CI]: 0.75 [0.62–0.91]; *p* = 3.4 × 10^−3^). Genetically predicted higher SBP was also associated with a higher probability of taking antihypertensive medication (*p* = 6.7 × 10^−8^). Genetically predicted smoking quantity was associated with lower AD risk (OR per ten cigarettes per day [95% CI]: 0.67 [0.51–0.89]; *p* = 6.5 × 10^−3^), although we were unable to stratify by smoking history; genetically predicted smoking initiation was not associated with AD risk (OR = 0.70 [0.37, 1.33]; *p* = 0.28). We saw no evidence of causal associations between glycemic traits, T2D, BMI, or educational attainment and risk of AD (all *p* > 0.1). Potential limitations of this study include the small proportion of intermediate trait variance explained by genetic variants and other implicit limitations of MR analyses.

**Conclusions:**

Inherited lifetime exposure to higher SBP is associated with lower AD risk. These findings suggest that higher blood pressure—or some environmental exposure associated with higher blood pressure, such as use of antihypertensive medications—may reduce AD risk.

## Abstract 2

Robert A. Scott and colleagues use genetic instruments to identify causal associations between known risk factors and Alzheimer's disease.

## Editors' Summary

**Background:**

Worldwide, about 44 million people have dementia, a group of brain degeneration disorders characterized by an irreversible decline in memory, communication, and other “cognitive” functions. Dementia mainly affects older people, and because people are living longer, experts estimate that more than 135 million people will have dementia by 2050. The most common form of dementia, which accounts for 60%–70% of cases, is Alzheimer disease (AD). The earliest sign of AD is often increasing forgetfulness. As the disease progresses, affected individuals gradually lose the ability to look after themselves, they may become anxious or aggressive, and they may have difficulty recognizing friends and relatives. People with late stage disease may lose control of their bladder and of other physical functions. At present, there is no cure for AD, although some of its symptoms can be managed with drugs. Most people with AD are initially cared for at home by relatives and other caregivers, but many affected individuals end their days in a care home or specialist nursing home.

**Why Was This Study Done?:**

Researchers are interested in identifying risk factors for AD, particularly modifiable risk factors, because if such risk factors exist, it might be possible to limit the predicted increase in future AD cases. Epidemiological studies (investigations that examine patterns of disease in populations) have identified several potential risk factors for AD, including hypertension (high blood pressure), obesity, smoking, and dyslipidemia (changes in how the body handles fats). However, epidemiological studies cannot prove that a specific risk factor causes AD. For example, people with hypertension might share another characteristic that causes both hypertension and AD (confounding) or AD might cause hypertension (reverse causation). Information on causality is needed to decide which risk factors to target to help prevent AD. Here, the researchers use “Mendelian randomization” to examine whether differences in several epidemiologically identified risk factors for AD have a causal impact on AD risk. In Mendelian randomization, causal associations are inferred from the effects of genetic variants (which predict levels of modifiable risk factors) on the outcome of interest. Because gene variants are inherited randomly, they are not prone to confounding and are free from reverse causation. So, if hypertension actually causes AD, genetic variants that affect hypertension should be associated with an altered risk of AD.

**What Did the Researchers Do and Find?:**

The researchers identified causal associations between potentially modifiable risk factors and AD risk by analyzing the occurrence of single nucleotide polymorphisms (SNPs, a type of gene variant) known to predict levels of each risk factor, in genetic data from 17,008 individuals with AD and 37,154 cognitively normal elderly controls collected by the International Genomics of Alzheimer’s Project. They report that genetically predicted higher systolic blood pressure (SBP; the pressure exerted on the inside of large blood vessels when the heart is pumping out blood) was associated with lower AD risk (and with a higher probability of taking antihypertensive medication). Predicted smoking quantity was also associated with lower AD risk, but there was no evidence of causal associations between any of the other risk factors investigated and AD risk.

**What Do These Findings Mean?:**

In contrast to some epidemiological studies, these findings suggest that hypertension is associated with lower AD risk. However, because genetically predicted higher SBP was also associated with a higher probability of taking antihypertensive medication, it could be that exposure to such drugs, rather than having hypertension, reduces AD risk. Like all Mendelian randomization studies, the reliability of these findings depends on the validity of several assumptions made by the researchers and on the ability of the SNPs used in the analyses to explain variations in exposure to the various risk factors. Moreover, because all the participants in the International Genomics of Alzheimer’s Project are of European ancestry, these findings may not be valid for other ethnic groups. Given that hypertension is a risk factor for cardiovascular disease, the researchers do not advocate raising blood pressure as a measure to prevent AD (neither do they advocate that people smoke more cigarettes to lower AD risk). Rather, given the strong association between higher SBP gene scores and the probability of exposure to antihypertensive treatment, they suggest that the possibility that antihypertensive drugs might reduce AD risk independently of their effects on blood pressure should be investigated as a priority.

**Additional Information:**

This list of resources contains links that can be accessed when viewing the PDF on a device or via the online version of the article at http://dx.doi.org/10.1371/journal.pmed.1001841.

- The UK National Health Service Choices website provides information (including personal stories) about Alzheimer disease
- The UK not-for-profit organization Alzheimer’s Society provides information for patients and carers about dementia, including personal experiences of living with Alzheimer disease
- The US not-for-profit organization Alzheimer’s Association also provides information for patients and carers about dementia and personal stories about dementia
- Alzheimer’s Disease International is the federation of Alzheimer disease associations around the world; it provides links to individual Alzheimer associations, information about dementia, and links to world Alzheimer reports
- MedlinePlus provides links to additional resources about Alzheimer disease (in English and Spanish)
- Wikipedia has a page on Mendelian randomization (note: Wikipedia is a free online encyclopedia that anyone can edit; available in several languages)
- A *PLOS Medicine* Research Article by Proitsi et al. describes a Mendelian randomization study that looked for a causal association between dyslipidemia and Alzheimer disease

## Introduction

Alzheimer disease (AD) prevalence is rising [1], further increasing the social and economic burden of this disease [2]. Epidemiological studies have aimed to identify potentially modifiable risk factors that could be targeted in preventive measures to reduce the incidence of AD. These include type 2 diabetes (T2D) and glycemic traits [3,4], hypertension [5], obesity [6], dyslipidemia [7], smoking [8], physical inactivity [5], depression [9], and low educational attainment [5]. It has been reported that approximately one-third of AD cases worldwide may be attributable to these risk factors [9]. However, this suggestion is predicated on these risk factors having causal effects on AD risk, which is currently uncertain [9]. Given the difficulties in implementing large-scale randomized trials of risk factor modification, alternative approaches are required to investigate the causality of associations and to prioritize the targets for which interventions may be most fruitful [10].

One method for estimating the causal effects of risk factors with known genetic determinants is Mendelian randomization (MR) [11]. The MR approach exploits the fact that genotypes are randomly assorted at meiosis, and are thus independent of conventional confounding factors and the disease process. Therefore, genetic variants associated with intermediate traits can be used to provide an unconfounded estimate of the causal association between the intermediate trait and disease outcome, unaffected by reverse causality. This is akin to a “genetically randomized trial.” For example, if body mass index (BMI) is causally associated with AD, genetic variants causing higher BMI should also be associated with higher risk of AD. However, if an observed BMI—AD association is not causal but is due to confounding or reverse causation, genetic variants causing higher BMI would not result in higher risk of AD. Here, we sought to estimate the causal effects of potentially modifiable risk factors on risk of AD using MR to inform the etiology of AD and the extent to which AD may be preventable by interventions targeting potentially modifiable risk factors.

## Methods

### Study Design

We performed MR analyses using single nucleotide polymorphisms (SNPs) with known associations with potentially modifiable AD risk factors. We used summary statistics from the International Genomics of Alzheimer’s Project (IGAP) [12], the largest genome-wide meta-analysis of AD reported to date, and individual genotype data from a large subset of IGAP to estimate the unconfounded association between each risk factor and AD risk. S1 Fig illustrates the study design.

### SNPs Associated with Alzheimer Disease Risk Factors

We identified SNPs that had genome-wide significant (*p <* 5 × 10^−8^) associations with each risk factor using the largest published genome-wide meta-analysis available in individuals of European ancestry. We identified 49 SNPs associated with T2D [13], 36 with fasting glucose [14], and ten with insulin resistance [14,15]. We identified 32 SNPs associated with BMI [16] and 25 associated with systolic blood pressure (SBP) [17]. Given the overlap of SNPs associated with systolic, diastolic, mean arterial, and pulse pressures, we focused on SBP, which had the largest number of associated SNPs [17,18]. We identified 74 SNPs associated with total cholesterol, 71 with high-density lipoprotein (HDL)–cholesterol, 58 with low-density lipoprotein (LDL)–cholesterol, and 40 with triglycerides [19]. We identified one SNP associated with smoking initiation (rs6265 in *BDNF*; *r* ^2^ = 0.74 with the *BDNF* BMI-associated variant), and three associated with smoking quantity in smokers [20]. We identified two SNPs associated with the probability of completing university and one associated with the number of years of education [21]. We show the SNPs and their associations with their relative traits in S1 Table. Where lead SNPs were not available, we selected a suitable proxy (*r* ^2^ > 0.8; except for rs4420638, where the best available proxy was rs6857 [*r* ^2^ = 0.46]), as detailed in S1 Table. Within each trait, no SNPs were in linkage disequilibrium (LD) (*r* ^2^ < 0.01). No SNPs have been reported to be associated with physical activity levels or depression at *p <* 5 × 10^−8^.

### Alzheimer Disease Genetic Data

IGAP is a large two-stage study based upon genome-wide association studies (GWASs) of AD in individuals of European ancestry [12]. In stage 1, IGAP used genotyped and imputed data on 7,055,881 SNPs to meta-analyze four previously published GWAS datasets consisting of 17,008 AD cases and 37,154 controls (full details in S1 Text). Further details on the original genetic discovery analyses, including information regarding recruitment and diagnostic assessment as well as analytical approaches to adjust for population structure, are provided in S1 Text or described in detail elsewhere [12]. We extracted individual SNP associations with AD from IGAP’s stage 1 results. Three SNPs (rs850303 for SBP; rs3177928 for total and LDL-cholesterol; rs645040 for triglycerides) were not available (S1 Table), so were excluded from analyses.

### Mendelian Randomization Analyses

We used estimated SNP—risk factor and SNP—AD associations to calculate estimates of each risk factor—AD association using an inverse-variance weighted combination of estimates from each SNP [22]. For continuous exposures (BMI, fasting glucose, insulin resistance, lipids, and SBP), we scaled MR estimates per standard deviation (SD) difference of the risk factor. Effect sizes on log-fasting insulin were used as weights for the insulin-resistance-associated variants. SDs were estimated from up to 10,445 (*N*_min_ = 9,963) middle-aged adults from the UK population-based Fenland study [23]. Causal estimates are thus presented per genetically predicted SD, and a log-linear association with odds of AD is implicit across the range of intermediate risk factor values. We scaled smoking quantity per ten cigarettes per day and scaled educational attainment per year of education. For binary exposures (T2D, smoking initiation, completing university), MR estimates are odds ratios (ORs) per genetically predicted unit difference in log-odds of having the relevant exposure. Overall, we included 302 non-overlapping SNPs. To minimize the possibility of pleiotropic associations influencing results, we performed sensitivity analyses excluding SNPs with a more significant association with AD than expected by chance (*p <* 0.05/302 = 0.00017), which excluded only four variants in total (S1 Table). Furthermore, we investigated the association of each variant with the risk factor relative to the magnitude of association with AD risk to further identify variants that appeared to be outliers and were candidates to be pleiotropic. As a further sensitivity analysis, for risk factors that showed evidence of a causal association with AD (*p <* 3.8 × 10^−3^), we also performed a “leave one out” analysis to further investigate the possibility that the causal association was driven by a single SNP.

We also performed MR analyses of risk factors that showed evidence of a causal association with AD (*p <* 3.8 × 10^−3^) using individual-level SNP data from studies in the Alzheimer’s Disease Genetics Consortium (ADGC) (cases = 10,079; controls = 9,613) [24] and the Genetic and Environmental Risk in AD (GERAD1) Consortium (cases = 3,146; controls = 1,224) [25], which account for 51% of the IGAP effective sample size (see S1 Text for a description of the ADGC and GERAD1 samples). We performed logistic regression analyses of the SNP-predicted AD association adjusting for study site, population substructure, age, and sex, again scaled per 1-SD difference in risk factor.

We created unweighted genetic scores based on the number of risk alleles for each SNP—risk factor association and investigated the association of these scores with a range of traits in up to 16,554 individuals from the EPIC-InterAct study [26] to check the assumption that the SNPs used in the MR analyses are not associated with potential confounders of exposure—AD associations. We standardized outcomes and included scores in linear regression models adjusted for age, sex, recruitment center, and subcohort status. We natural-log-transformed triglyceride levels before standardization. We investigated the association of the SBP-associated variants with both SBP and diastolic blood pressure (DBP). We did not adjust observed blood pressure values for antihypertensive usage. We used logistic regression to determine associations with the probability of being physically active, being a smoker, or taking antihypertensive medications, and included covariates as above. The distribution of the SBP risk score in the EPIC-InterAct study [26] is shown in S2 Fig.

## Results

**Fig 1:**
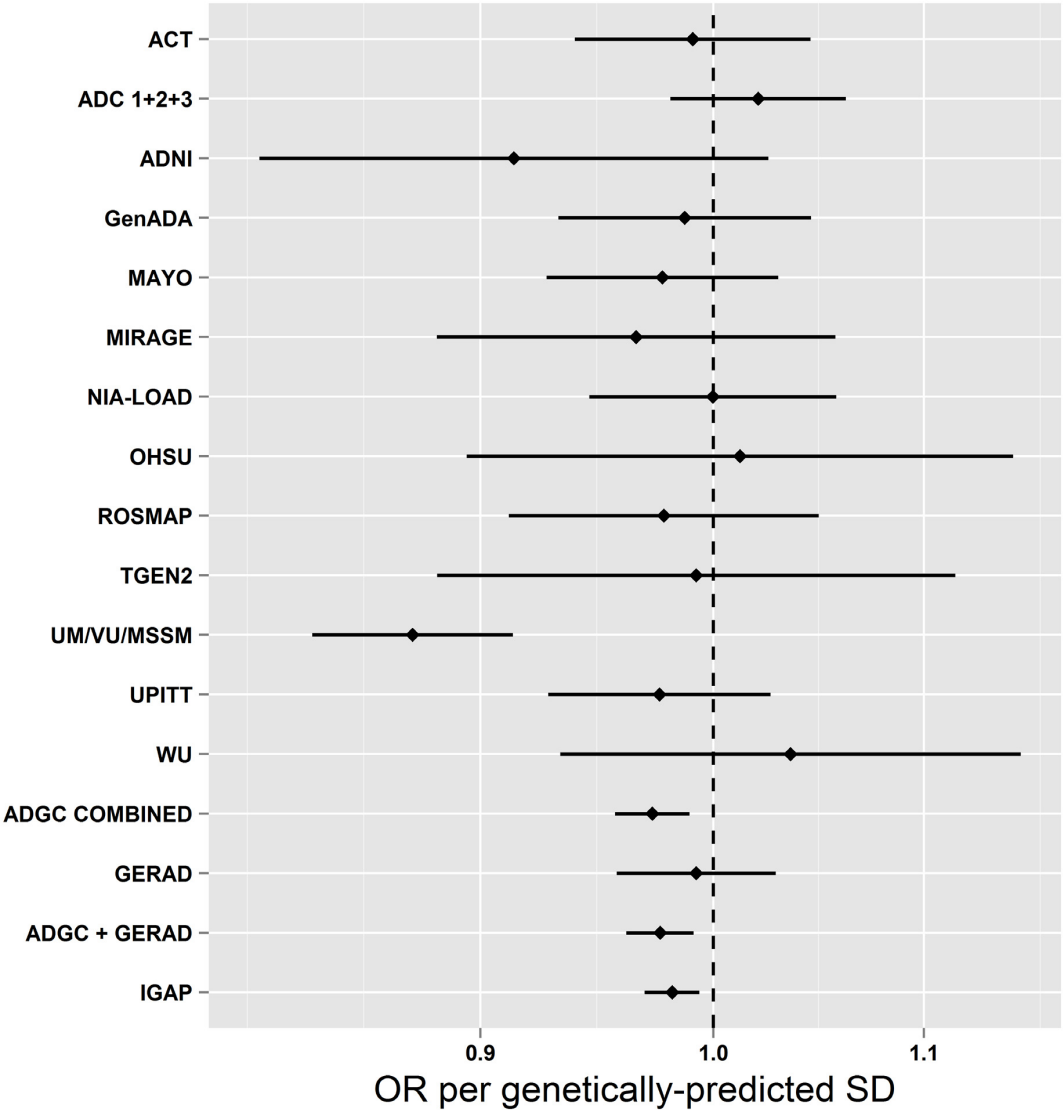
Mendelian randomization estimates of the association of systolic blood pressure with AD in individual ADGC studies and overall in ADGC, GERAD1, and IGAP. This figure shows MR estimates for the association of SBP-associated variants with AD in each of the participant studies in ADGC [24] and in GERAD1 [25] using individual SNP-level data compared to that observed in IGAP [12] using summary-level data. See S1 Text (supplemental results) for individual study name abbreviations.

Table 1 shows the estimated associations of each genetically predicted risk factor with AD from MR analysis using a large-scale international investigation of the genetic basis of AD risk in 17,008 individuals with AD and 37,154 controls. We observed evidence for a causal association between genetically predicted SBP and AD risk. A genetically predicted 1-SD (15.4 mm Hg) higher SBP was associated with lower risk of AD (OR [95% CI]: 0.75 [0.62–0.91]; *p =* 3.4 × 10^−3^). We examined each of the SBP SNPs to investigate if particular SNPs were driving the association with AD, but observed no obvious outliers (S3 Fig). Furthermore, when we performed all 24 permutations of the “leave one out” analysis, all SNP sets showed consistent evidence of causality (OR per SD of SBP [95% CI] ranged from 0.72 [0.59–0.87] to 0.78 [0.64–0.95]). Individual SNP associations with AD are shown in S1 Table. We also performed analyses on a subset of the overall sample using individual-level SNP data from ADGC and GERAD1, which showed results similar to those observed using the inverse-variance weighted approach (OR [95% CI]: 0.69 [0.55–0.85]; *p =* 2.0 × 10^−3^; Fig 1). We saw no evidence of heterogeneity between individual studies (*p =* 0.33).

**Table 1:** Estimated associations of each genetically predicted risk factor with Alzheimer disease.

Trait	Scaling of OR	Number of SNPs	Overall Results		Sensitivity Analyses*	
			OR (95% CI)	*p*-Value	OR (95% CI)	*p*-Value
BMI	1 SD (4.81 kg/m^2^)	32	0.99 (0.80−1.19)	0.779	1.00 (0.82−1.22)	0.97
T2D	1 unit higher log-odds	49	1.02 (0.97−1.07)	0.535		
Fasting glucose	1 SD (0.65 mmol/l)	36	1.12 (0.97−1.30)	0.112	1.19 (1.03−1.37)	0.02
Insulin resistance	1 SD log-FI (0.60 log-pmol/l)	10	1.32 (0.88−1.98)	0.177		
SBP	1 SD (15.4 mm Hg)	24	0.75 (0.62−0.91)	3.4 × 10^−3^		
Total cholesterol	1 SD (1.03 mmol/l)	73	1.94 (1.79−2.10)	3.1 × 10^−56^	1.04 (0.95−1.13)	0.84
HDL-cholesterol	1 SD (0.41 mmol/l)	71	0.75 (0.69−0.82)	1.0 × 10^−11^	1.01 (0.93−1.09)	0.87
LDL-cholesterol	1 SD (0.91 mmol/l)	57	2.31 (2.12−2.50)	3.0 × 10^−87^	1.07 (0.98−1.17)	0.14
Triglycerides	1 SD (0.83 mmol/l)	39	0.96 (0.87−1.07)	0.482		
Smoking initiation	1 unit higher log-odds	1	0.70 (0.37−1.33)	0.278		
Smoking quantity	10 cigarettes/day	3	0.67 (0.51−0.89)	6.5 × 10^−3^		
Completing university	1 unit higher log-odds	2	0.95 (0.67−1.34)	0.752		
Length of education	1 year of education	1	0.71 (0.48−1.06)	0.097		

*Sensitivity analyses exclude SNPs where *p* < 0.00017 (0.05/302 unique SNPs) for AD.

log-FI, log-fasting insulin.

**Fig 2:**
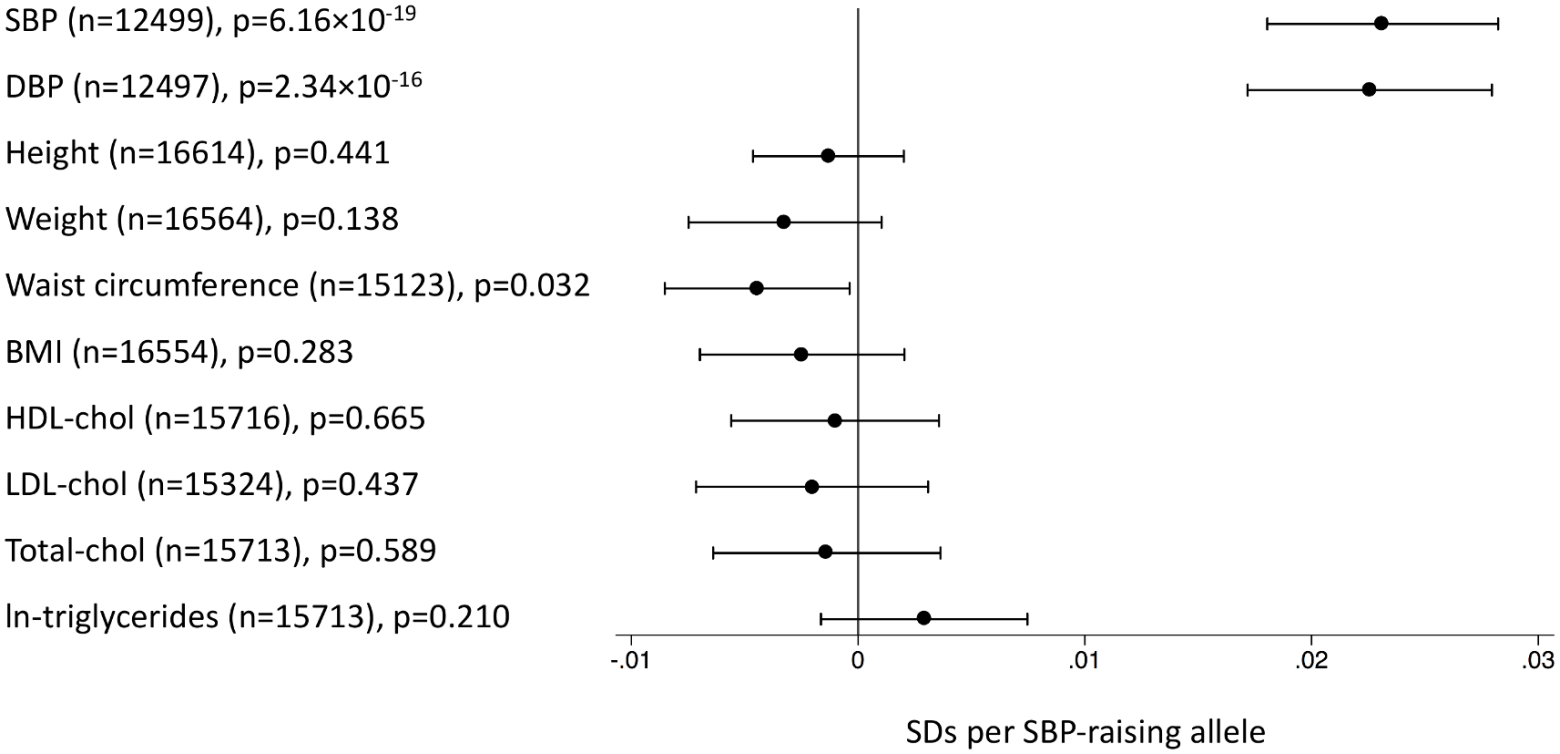
Associations of the systolic blood pressure genetic score with quantitative traits in the EPIC-InterAct study. This figure shows the investigation of pleiotropic associations of genetic score for SBP with quantitative traits in the EPIC-InterAct study [26]. Effect sizes are expressed in SDs per SBP-raising allele. Analyses were adjusted for age, sex, center of recruitment, and subcohort status.

**Fig 3:**
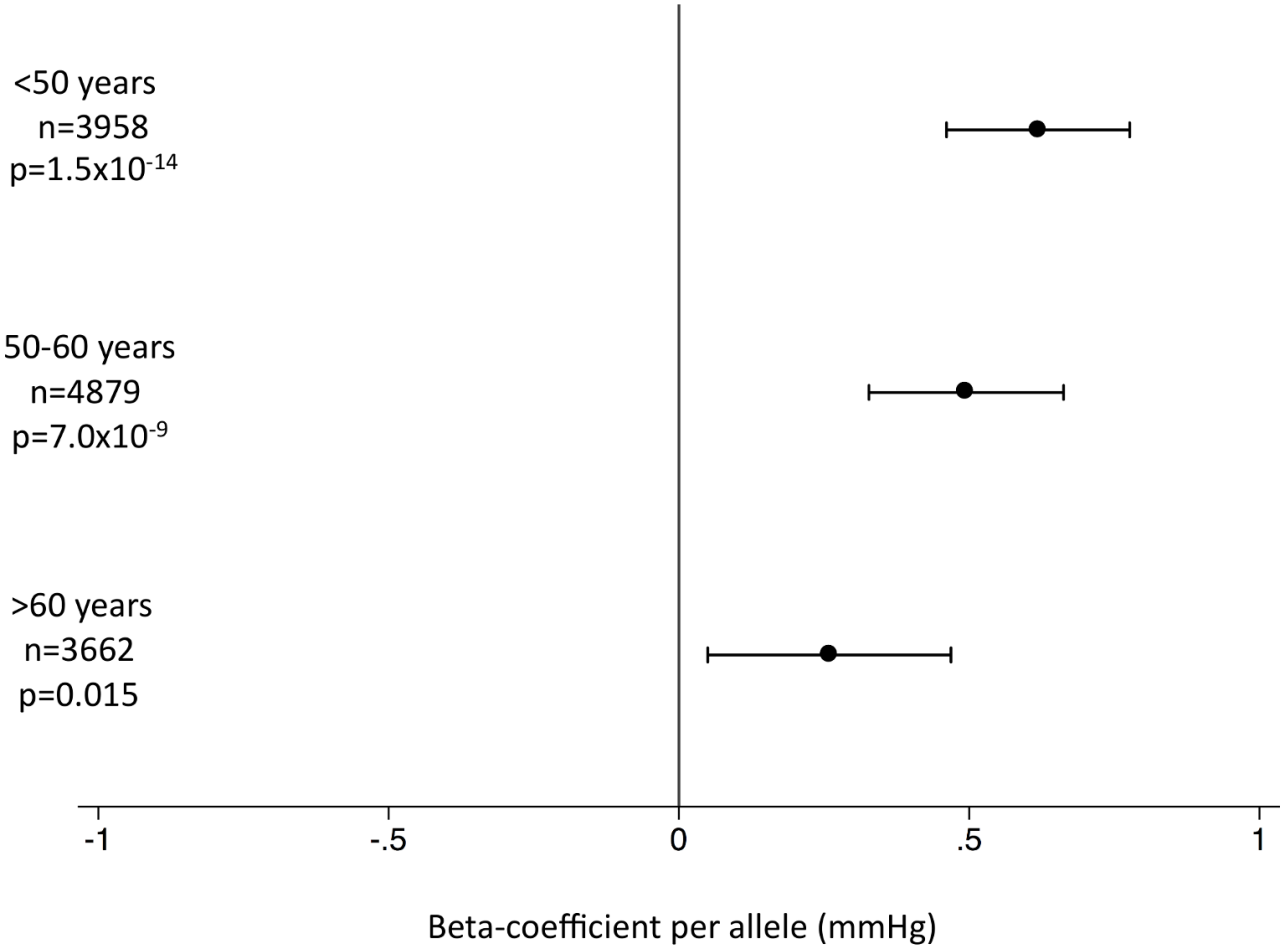
Association of the systolic blood pressure genetic score with systolic blood pressure by age stratum in the EPIC-InterAct subcohort. This figure shows the association between the genetic score for SBP and SBP in the EPIC-InterAct study by age stratum [26]. Analyses were adjusted for sex, center of recruitment, and subcohort status.

**Fig 4:**
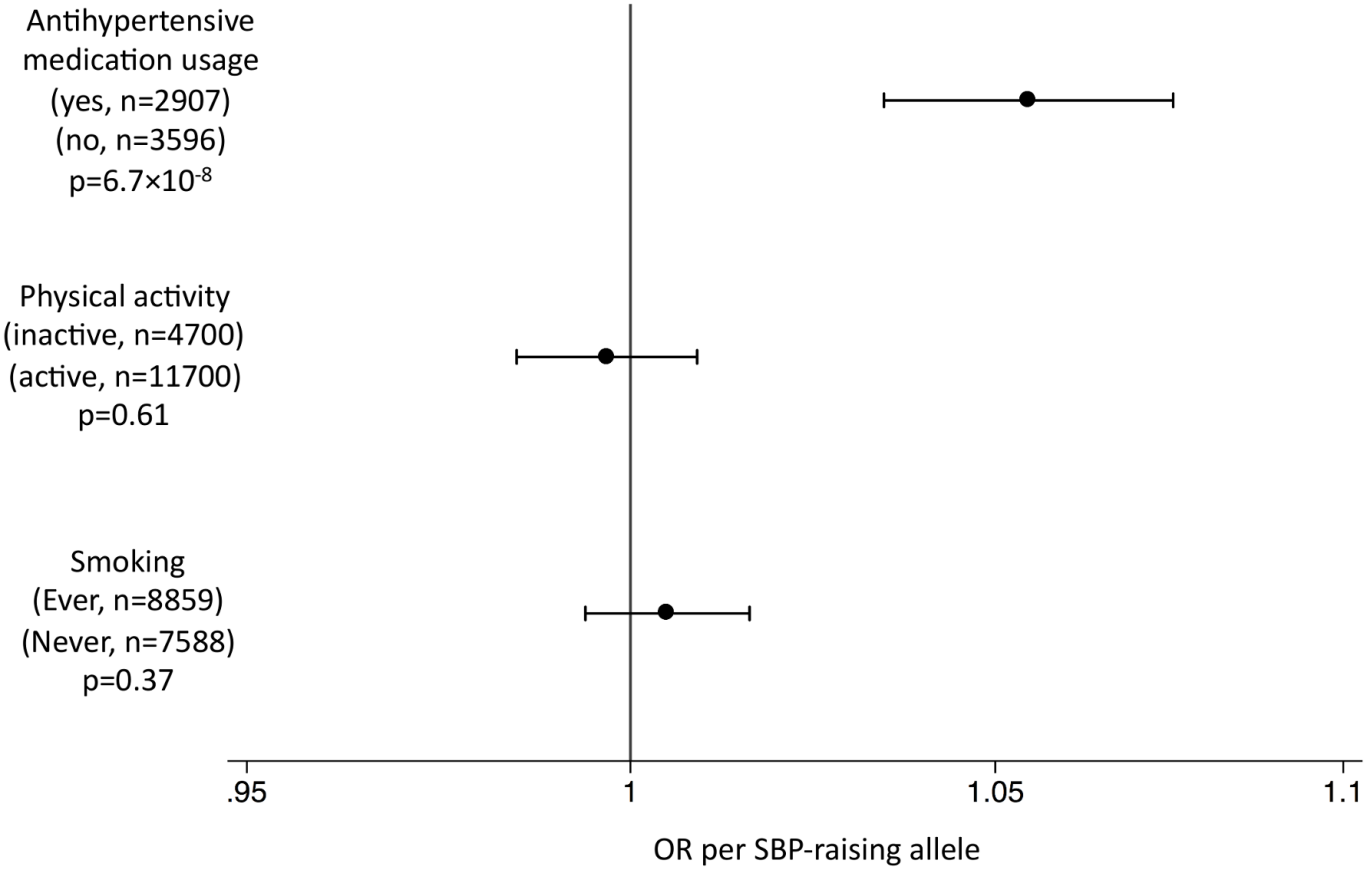
Associations of the systolic blood pressure genetic score with binary outcomes in the EPIC-InterAct study. This figure shows the investigation of pleiotropic associations of the genetic score for SBP with binary outcomes in the EPIC-InterAct study [26]. The OR per SBP-raising allele is shown.

In the EPIC-InterAct study, the unweighted SBP genetic score was strongly associated with SBP and DBP overall (Fig 2) and in all age groups (Fig 3) (*p <* 0.015 for SBP; *p <* 0.002 for DBP). We did not observe associations of the SBP score with other potentially confounding variables in the EPIC-InterAct study (Fig 2). The unweighted SBP genetic score was associated with a higher probability of taking antihypertensive medication (OR [95% CI]: 1.05 [1.03–1.08]; *p =* 6.7 × 10^−8^) but not with the probability of being physically active or being a smoker (Fig 4). Forty-nine percent of the individuals in the highest quartile of the unweighted SBP genetic score reported taking antihypertensive medication compared to 39% in the lowest quartile.

We found strong associations between genetically predicted total, LDL-, and HDL-cholesterol and AD (Table 1). Each of these SNP sets included rs6857 near *APOE*, which is strongly associated with AD risk (OR: 3.2; *p =* 2.5 × 10^−575^) [12] (S4 and S5 Figs) and which was a very clear outlier when we compared effect sizes on lipids against effect sizes on AD (S6–S11 Figs). Two of these SNP sets also included rs1883025 from *ABCA1*, which was associated with AD at a significance level beyond that expected by chance (OR: 1.07; *p =* 1.0 × 10^−4^) [12] and which is in a gene previously implicated in association with AD [27]. After sensitivity analyses excluding these potentially pleiotropic SNPs, we saw no evidence for causal associations between lipid fractions and AD risk (Table 1).

We found no evidence to support causal associations between BMI and AD (OR per SD of BMI [95% CI]: 0.99 [0.80–1.19]; *p =* 0.78), fasting glucose (OR [95% CI]:1.12 [0.97–1.30]; *p =* 0.11), insulin resistance (OR [95% CI]: 1.32 [0.89–1.97]; *p =* 0.17), or T2D (OR [95% CI]: 1.01 [0.96–1.07]; *p =* 0.57) (Table 1). S4 Fig shows the associations with AD of the SNPs included in all genetic analyses compared to those expected by chance. Other than rs6857 near *APOE*, the most significant associations with AD were observed for rs11039149 (*p =* 3.7 × 10^−6^) in the fasting glucose SNPs and rs3817334 (*p =* 9.3 × 10^−5^) in the BMI SNPs (see S1 Table and S5 Fig). These SNPs are both in LD (*r* ^2^ = 0.58 and *r* ^2^ = 0.33, respectively) with a genome-wide significant association signal for AD in *CELF1* [12]. After excluding these variants from their respective SNP sets, BMI results were unchanged (Table 1). However, for fasting glucose, following the removal of rs11039149 near *MADD*, there was a suggestion of an association between higher glucose and higher AD risk (OR [95% CI]: 1.19 [1.03–1.37]; *p =* 0.02).

We found no evidence to support causal associations between smoking initiation and AD (OR [95% CI]: 0.70 [0.37–1.33]; *p =* 0.28). We did find an association between genetically predicted higher smoking quantity and lower AD (OR per ten cigarettes/day [95% CI]: 0.67 [0.51–0.89]; *p =* 6.5 × 10^−3^). We did not have smoking behavior data for IGAP to obtain estimates for the association with AD among smokers and non-smokers. The SNP with the strongest association with smoking quantity [20] was nominally associated with AD risk (rs1051730: OR of AD per smoking-quantity-raising allele [95% CI]: 0.96 [0.93–0.99]; *p =* 0.01), while the others were not (S12 Fig). We saw no association between AD risk and either university completion (OR [95% CI]: 0.95 [0.67–1.34]; *p =* 0.75) or years of education (OR [95% CI]: 0.71 [0.48–1.06]; *p =* 0.10) (Table 1).

## Discussion

The potential of risk factor modifications to impact upon AD incidence depends entirely on causal links between the risk factors and AD. Using genetic variants associated with risk factors for AD in a very large consortium of well-characterized research participants, we found evidence for an association between genetically inherited higher levels of blood pressure and lower AD risk.

Hypertension has been implicated as a risk factor for AD [5]. However, uncertainties remain over the nature of the association, perhaps complicated by misclassification of AD with other forms of dementia, or the age of study participants [28]. While previous studies have suggested that high blood pressure in midlife is associated with higher AD risk [29,30], other studies have indicated that high blood pressure in late life may be protective against AD [31,32]. We found that genetically inherited higher SBP levels are associated with lower risk of AD (Table 1). Previous studies have suggested that hypotension may indeed be a risk factor for AD, particularly in the elderly [33], potentially via resultant cerebral hypoperfusion [34]. The unweighted SBP gene score was associated with higher SBP levels across the adult lifespan (Fig 3). It should be noted that the SNPs associated with SBP overlap extensively with those associated with DBP [17] as well as with pulse pressure [18], so we were unable to distinguish between individual components of blood pressure. A recent meta-analysis of prospective studies suggested that a 10-mm Hg higher SBP was associated with a protective relative risk of 0.95 (95% CI: 0.91–1.00) for AD [28]. Scaling our results to a genetically predicted 10-mm Hg difference in SBP would result in an OR of 0.83 (95% CI: 0.73–0.94) for AD. Clearly, given that blood pressure is a major risk factor for cardiovascular disease [35], one would not advocate raising blood pressure as a preventive strategy, yet these findings offer intriguing etiological insight.

We also found that genetically predicted higher SBP was associated with a higher probability of being on antihypertensive medication (Fig 4). There is considerable interest in the role of antihypertensives in dementia, and while findings are equivocal [36], recent studies have suggested a possible protective effect of antihypertensive therapy on AD risk [37], potentially with heterogeneity of effect by therapeutic class [38], suggesting that any effect on AD risk may not be entirely attributable to the lowering of blood pressure, but potentially to other mechanisms. The unweighted SBP gene score was strongly associated with observed SBP in the EPIC-InterAct study, ignoring any SBP-lowering effects of antihypertensive medications. Thus, if antihypertensive medications are indeed protective and confound the association between genetically predicted SBP and AD, their effect on AD risk is likely to be independent of their effect on SBP, as the SBP-associated variants have a strong association with SBP regardless of the higher prevalence of treatment with antihypertensive medication. While the null association between genetically predicted lipid levels and AD risk reflects the equivocal findings from trials of statins and cognitive decline [39], our results suggest the imperative need for further investigation of the possibility that antihypertensive medications may reduce AD risk independently of their effects on blood pressure. Future MR analyses stratified by antihypertensive treatments would be desirable to more precisely estimate the magnitude of the causal effect of higher BP on AD risk, but will be difficult to carry out using existing data due to the time-varying nature of antihypertensive treatments across the life course, and the non-availability of data on lifetime medication usage in most studies.

We also observed an association between AD and smoking quantity (Table 1). Early reports implicated smoking as protective for AD [40], potentially via a neuroprotective effect of nicotine [41]. However, this association may be due to differential survival bias [42], and a recent meta-analysis of prospective studies implicates smoking as a risk factor for AD, showing current smokers as being at higher risk of AD than never smokers [8]. One smoking-quantity-related SNP was associated with AD (*p =* 0.01). This SNP is in the gene *CHRNA3* from the nicotinic receptor gene cluster *CHRNA5-CHRNA3-CHRNB4*. Given the putative actions of nicotine, variants in this locus may confer neuroprotective effects by influencing nicotinic receptor function [41,43]. Thus, altered nicotinic receptor function may underlie the MR association between smoking quantity and AD risk. The ideal study would perform MR analyses stratified by smoking status [44], particularly if sensitivity analyses could exclude variants in nicotinic receptor genes. Such analyses would address the causality of smoking as a risk factor, and offer valuable insight into nicotine’s role in the etiology of AD [41,43]. Since smoking is a major cause of global disease burden [45], increasing knowledge of the role of nicotine in the etiology of AD may prove to be the more actionable insight.

Our findings for total, LDL-, and HDL-cholesterol are not consistent with a causal effect of major lipid fractions on AD risk, as previously suggested in a smaller study [46]. Rather, the well-established association of *APOE* haplotypes with AD risk [47] implicates *APOE* itself as a key causal factor in the etiology of AD. Indeed a recent GWAS of plasma APOE levels identified only genetic variants in *APOE*, and not those in other lipid loci, as being associated with APOE levels at genome-wide significance [48]. When we compared the effect sizes for the effects of SNPs on major lipids relative to the magnitude of their association with AD, the *APOE* variant was a very clear outlier (S6–S11 Figs).

We did not find evidence consistent with a causal role for the other potentially modifiable risk factors we evaluated (Table 1). In our sensitivity analysis that excluded the potentially pleiotropic variant near *MADD*, genetically predicted higher fasting glucose was nominally associated with higher AD risk. While these results are consistent with the notion that higher blood glucose may be causally related to AD risk [4], the borderline significance warrants a cautious interpretation.

A limitation of the MR approach is the limited strength of the SNPs to explain variation in the intermediate traits, restricting statistical power. This is particularly true when findings are null, where narrow confidence intervals are important to aid robust inference. For example, while we saw no evidence to support causal roles for BMI, fasting glucose, or insulin resistance in AD (all *p* > 0.1), confidence intervals allow for an almost 20% higher AD risk per 1-SD difference in BMI, a 30% higher AD risk per 1-SD difference in fasting glucose, and an almost 100% higher AD risk per 1-SD difference in log-fasting insulin (Table 1). Thus, improving the intermediate trait variance explained by the instrumental variables by further genetic discovery efforts will improve the precision of MR analyses. Likewise, ever larger AD GWASs will further narrow confidence intervals around MR estimates. The association of genetically predicted blood pressure with AD risk remained after Bonferroni correction for the 13 individual SNP sets we tested (0.05/13 = 3.8 × 10^−3^), although the association of the smoking-associated variants did not. However, we consider this a conservative correction, given the correlation between the intermediate risk factors. We cannot exclude the possibility that the protective associations of blood pressure with AD arise as a result of differential survival bias, but the consistency of the observations across both prospective and cross-sectional studies of AD makes this less likely (Fig 1), as does the absence of similar MR associations for other major vascular risk factors (Table 1).

The main data source for this study is the summary statistics from IGAP, the largest genome-wide meta-analysis of AD reported to date [12]. Since all participants in IGAP are of European ancestry, the results of this study are not necessarily valid for other ethnic groups.

In conclusion, we found associations between genetically predicted higher SBP and lower AD risk. This finding is contrary to the notion that societal interventions to lower blood pressure will reduce the incidence of AD. However, since there is a strong association between higher SBP gene scores and exposure to antihypertensive treatments, there is a need to evaluate the possible protective role of some of these substances against AD, independent of their effects on blood pressure.

## Supporting Information

S1 Checklist STROBE checklist. (DOCX)

S1 Fig Illustration of the study design. (TIF)

S2 Fig Distribution of the systolic blood pressure genetic risk score in the EPIC-InterAct study. *n* = 16,691.
(TIF)

S3 Fig Estimated associations of SNPs with AD (ln-ORs and 95% CIs) from IGAP [12] against their estimated associations with SBP from [17]. (TIF)

S4 Fig QQ plot of SNP associations with Alzheimer disease. SNPs from all scores (*N*_unique_ = 302) were LD-pruned and duplicates were removed to leave the 269 variants shown here. The SNP near *APOE* (rs6857) had a *p*-value of 2.5 × 10^−575^, which was truncated to 10^−30^ for display on the figure.
(TIF)

S5 Fig QQ plot of SNP associations with Alzheimer disease excluding the *APOE* allele. SNPs from all scores (*N*_unique_ = 302) were LD-pruned and duplicates were removed to leave 269 variants. For the present plot, the SNP near *APOE* (rs6857, *p* = 2.5 × 10^−575^) was excluded (see S4 Fig).
(TIF)

S6 Fig Estimated associations of SNPs with AD (ln-ORs and 95% CIs) from IGAP [12] against their estimated associations with total cholesterol from [19] including the *APOE* allele. (TIF)

S7 Fig Estimated associations of SNPs with AD (ln-ORs and 95% CIs) from IGAP [12] against their estimated associations with total cholesterol from [19] excluding the *APOE* allele. (TIF)

S8 Fig Estimated associations of SNPs with AD (ln-ORs and 95% CIs) from IGAP [12] against their estimated associations with HDL-cholesterol from [19] including the *APOE* allele. (TIF)

S9 Fig Estimated associations of SNPs with AD (ln-ORs and 95% CIs) from IGAP [12] against their estimated associations with HDL-cholesterol from [19] excluding the *APOE* allele. (TIF)

S10 Fig Estimated associations of SNPs with AD (ln-ORs and 95% CIs) from IGAP [12] against their estimated associations with LDL-cholesterol from [19] including the *APOE* allele. (TIF)

S11 Fig Estimated associations of SNPs with AD (ln-ORs and 95% CIs) from IGAP [12] against their estimated associations with LDL-cholesterol from [19] excluding the *APOE* allele. (TIF)

S12 Fig Estimated associations of SNPs with AD (ln-ORs and 95% CIs) from IGAP [12] against their estimated associations with smoking quantity derived from the Tobacco and Genetics Consortium GWAS [20]. *Note that the effect sizes for the effects of SNPs on smoking quantity were estimated in current smokers only, while the associations with AD are not stratified by smoking status.
(TIF)

S1 Table SNPs associated with putative Alzheimer disease risk factors. SNPs included in each risk score, their associations estimated by the relevant consortium, and their association with AD (IGAP [12]). Proxies are marked by an asterix in the leftmost column. The effect allele frequency was based on data from the EPIC-InterAct study [26]. Double asterisks indicate that frequency information was not available in the EPIC-InterAct study, and were obtained from European 1000 Genomes samples.
(XLSX)

S1 Text Supplemental methods, supplemental results, and acknowledgments. (DOCX)

## Abbreviations

AD: Alzheimer disease
ADGC: Alzheimer’s Disease Genetics Consortium
BMI: body mass index
DBP: diastolic blood pressure
GERAD1: Genetic and Environmental Risk in AD
GWAS: genome-wide association study
HDL: high-density lipoprotein
IGAP: International Genomics of Alzheimer’s Project
LD: linkage disequilibrium
LDL: low-density lipoprotein
MR: Mendelian randomization
OR: odds ratio
SD: standard deviation
SNP: single nucleotide polymorphism
SBP: systolic blood pressure
T2D: type 2 diabetes
