# Supplementary material for: Associations between Potentially Modifiable Risk Factors and Alzheimer Disease: A Mendelian Randomization Study
Source: PLoS Med. 2015 Jun 16;12(6):e1001841. doi: 10.1371/journal.pmed.1001841 (PMC4469461; doi:10.1371/journal.pmed.1001841)
Supplement: S1 Checklist — (DOCX) [file pmed.1001841.s001.docx]

STROBE Statement—checklist of items that should be included in reports of observational studies

|  | Item No | Recommendation |
| --- | --- | --- |
| **Title and abstract** | 1 | (*a*) Indicate the study’s design with a commonly used term in the title or the abstract  Reply: See title: “Associations between potentially modifiable risk factors and Alzheimer’s disease: A Mendelian randomization study” |
|  |  | (*b*) Provide in the abstract an informative and balanced summary of what was done and what was found  Reply: Done |
| Introduction | | |
| Background/rationale | 2 | Explain the scientific background and rationale for the investigation being reported  Reply: Done |
| Objectives | 3 | State specific objectives, including any prespecified hypotheses  Reply: Done |
| Methods | | |
| Study design | 4 | Present key elements of study design early in the paper  Reply: See methods section, first paragraph: “We performed MR analyses using single nucleotide polymorphisms (SNPs) with known associations with potentially modifiable AD risk factors. We used summary statistics from the International Genomics of Alzheimer's Project (IGAP) [12], the largest genome-wide meta-analysis of AD reported to date, and individual genotype data from a large subset of IGAP, to estimate the unconfounded association between each risk factor and AD risk. S1 Fig. illustrates the study design.” |
| Setting | 5 | Describe the setting, locations, and relevant dates, including periods of recruitment, exposure, follow-up, and data collection.  Reply: See methods section, third paragraph: “IGAP is a large two-stage study based upon genome-wide association studies (GWAS) of AD from individuals of European ancestry [12]. In stage 1, IGAP used genotyped and imputed data on 7055881 SNPs to meta-analyse four previously-published GWAS datasets consisting of 17008 AD cases and 37154 controls (full details in S1 Text). Further details on the original genetic discovery analyses, including information regarding recruitment and diagnostic assessment as well as analytical approaches to adjust for population structure are provided in S1 Text and described in detail elsewhere [12].” |
| Participants | 6 | (*a*) *Cohort study*—Give the eligibility criteria, and the sources and methods of selection of participants. Describe methods of follow-up  *Case-control study*—Give the eligibility criteria, and the sources and methods of case ascertainment and control selection. Give the rationale for the choice of cases and controls  Reply: This information is given in the supporting text (see paragraph entitled: “Alzheimer’s disease genetic data”).  *Cross-sectional study*—Give the eligibility criteria, and the sources and methods of selection of participants |
|  |  | (*b*) *Cohort study*—For matched studies, give matching criteria and number of exposed and unexposed  *Case-control study*—For matched studies, give matching criteria and the number of controls per case  Reply: This information is given in the supporting text (see paragraph entitled: “Alzheimer’s disease genetic data”). |
| Variables | 7 | Clearly define all outcomes, exposures, predictors, potential confounders, and effect modifiers. Give diagnostic criteria, if applicable  Reply: This information is provided in the methods section (Paragraph named: “Mendelian randomization analyses”) |
| Data sources/ measurement | 8* | For each variable of interest, give sources of data and details of methods of assessment (measurement). Describe comparability of assessment methods if there is more than one group  Reply: This information is provided in the methods section (Paragraph named: “Mendelian randomization analyses”) |
| Bias | 9 | Describe any efforts to address potential sources of bias  Reply: This information is provided in the methods section (Paragraph named: “Mendelian randomization analyses”) |
| Study size | 10 | Explain how the study size was arrived at  Reply: This information is provided in the methods section (Paragraph named: “Mendelian randomization analyses) |
| Quantitative variables | 11 | Explain how quantitative variables were handled in the analyses. If applicable, describe which groupings were chosen and why  Reply: This information is provided in the methods section (Paragraph named: “Mendelian randomization analyses”) |
| Statistical methods | 12 | (*a*) Describe all statistical methods, including those used to control for confounding  Reply: This information is provided in the methods section (Paragraph named: “Mendelian randomization analyses”) |
|  |  | (*b*) Describe any methods used to examine subgroups and interactions  Reply: Not applicable |
|  |  | (*c*) Explain how missing data were addressed  Reply: this information is provided in the methods section (Paragraph named: “SNPs associated with putative AD risk factors”): “Where lead SNPs were not available, we selected a suitable proxy (r^2^>0.8; except for rs4420638, where the best available proxy was rs6857 (r^2^=0.46)) as detailed in S1 Table. Within each trait, no SNPs were in LD (R^2^<0.01). No SNPs have been reported to be associated with physical activity levels or depression at p<5x10^-8^.” |
|  |  | (*d*) *Cohort study*—If applicable, explain how loss to follow-up was addressed  *Case-control study*—If applicable, explain how matching of cases and controls was addressed  Not applicable  *Cross-sectional study*—If applicable, describe analytical methods taking account of sampling strategy |
|  |  | (*e*) Describe any sensitivity analyses  Reply: This information is provided in the methods section (Paragraph named: “Mendelian randomization analyses”): “To minimize the possibility of pleiotropic associations influencing results, we performed sensitivity analyses excluding SNPs with a more significant association with AD than expected by chance (p<0.05/302=0.00017), which excluded only 4 variants in total (S1 Table). Furthermore, we investigated the association of each variant with the risk factor relative to the association with AD risk to further identify variants, which appeared to be outliers and were candidates to be pleiotropic. As a further sensitivity analysis, for risk factors, which showed evidence of a causal association with AD (p<3.8x10^-3^), we also performed a “leave one out” analysis to investigate the possibility that the causal association was driven by a single SNP”. |

| Results | | |
| --- | --- | --- |
| Participants | 13* | (a) Report numbers of individuals at each stage of study—eg numbers potentially eligible, examined for eligibility, confirmed eligible, included in the study, completing follow-up, and analysed  Reply: Reply: This information is given in the supporting text (see paragraph entitled: “Alzheimer’s disease genetic data”). |
|  |  | (b) Give reasons for non-participation at each stage  Reply: Not applicable |
|  |  | (c) Consider use of a flow diagram  Reply: A flow diagram has been made and included in the submission (S1 Fig.) |
| Descriptive data | 14* | (a) Give characteristics of study participants (eg demographic, clinical, social) and information on exposures and potential confounders  Reply: Not applicable |
|  |  | (b) Indicate number of participants with missing data for each variable of interest  Reply: Not applicable |
|  |  | (c) *Cohort study*—Summarise follow-up time (eg, average and total amount)  Reply: Not applicable |
| Outcome data | 15* | *Cohort study*—Report numbers of outcome events or summary measures over time |
|  |  | *Case-control study—*Report numbers in each exposure category, or summary measures of exposure  Reply: See S1 table. |
|  |  | *Cross-sectional study—*Report numbers of outcome events or summary measures |
| Main results | 16 | (*a*) Give unadjusted estimates and, if applicable, confounder-adjusted estimates and their precision (eg, 95% confidence interval). Make clear which confounders were adjusted for and why they were included  Reply: All results are provided as requested |
|  |  | (*b*) Report category boundaries when continuous variables were categorized  Reply: All results are provided as requested |
|  |  | (*c*) If relevant, consider translating estimates of relative risk into absolute risk for a meaningful time period  Reply: Not applicable – see response to reviewers |
| Other analyses | 17 | Report other analyses done—eg analyses of subgroups and interactions, and sensitivity analyses  Reply: See results section, first paragraph: “We also performed analyses on a subset of the overall sample using individual-level SNP data from ADGC and GERAD which showed similar results to those observed using the inverse-variance-weighted approach (OR = 0.69 [0.55-0.85]; p=2.0x10^-3^; Fig. 1). We saw no evidence of heterogeneity between individual studies (p=0.33).” |
| Discussion | | |
| Key results | 18 | Summarise key results with reference to study objectives  Reply: See discussion section, first paragraph: “The potential of risk factor modifications to impact AD incidence depends entirely on causal links between the risk factors and AD. Using genetic variants associated with risk factors for AD in a very large consortium of well-characterized research participants, we found evidence for an association between genetically inherited higher levels of blood pressure and lower AD risk.” |
| Limitations | 19 | Discuss limitations of the study, taking into account sources of potential bias or imprecision. Discuss both direction and magnitude of any potential bias  Reply: See discussion section, seventh paragraph: “A limitation of the MR approach is the limited strength of the SNPs to explain variation in the intermediate traits, restricting statistical power. This is particularly true when findings are null, where narrow confidence intervals are important to aid robust inference. For example, while we saw no evidence to support causal roles for BMI, fasting glucose or fasting insulin in AD (all p-values > 0.1), confidence intervals allow for almost 20% higher AD risk per-SD of BMI, 30% higher per-SD of fasting glucose, and almost 100% higher per-SD of fasting insulin (Table 1). Thus, improving the intermediate trait variance explained by the instrumental variables by further genetic discovery efforts will improve the precision of MR analyses.” |
| Interpretation | 20 | Give a cautious overall interpretation of results considering objectives, limitations, multiplicity of analyses, results from similar studies, and other relevant evidence  Reply: See discussion section, last paragraph: “In conclusion, we found associations between genetically-predicted higher SBP and lower AD risk. This finding is contrary to the notion that societal interventions to lower blood pressure will reduce the incidence of AD. However, since there is a strong association between higher SBP gene scores and exposure to antihypertensive treatments, there is a need to evaluate the possible protective role of some of these substances against AD, independent of their effects on blood pressure.” |
| Generalisability | 21 | Discuss the generalisability (external validity) of the study results  Reply: See discussion section, second-to-last paragraph: “The main data source for this study is the summary statistics from IGAP, the largest genome-wide meta-analysis of AD reported to date [12]. Since all participants in IGAP are of European ancestry, the results of this study are not necessarily valid for other ethnic groups.” |
| Other information | | |
| Funding | 22 | Give the source of funding and the role of the funders for the present study and, if applicable, for the original study on which the present article is based  Reply: This information is provided in the designated boxes on the submission platform as requested by the editorial office. |

*Give information separately for cases and controls in case-control studies and, if applicable, for exposed and unexposed groups in cohort and cross-sectional studies.

**Note:** An Explanation and Elaboration article discusses each checklist item and gives methodological background and published examples of transparent reporting. The STROBE checklist is best used in conjunction with this article (freely available on the Web sites of PLoS Medicine at http://www.plosmedicine.org/, Annals of Internal Medicine at http://www.annals.org/, and Epidemiology at http://www.epidem.com/). Information on the STROBE Initiative is available at www.strobe-statement.org.
