# Supplementary figures and images for: Associations between Potentially Modifiable Risk Factors and Alzheimer Disease: A Mendelian Randomization Study
Source: PLoS Med. 2015 Jun 16;12(6):e1001841. doi: 10.1371/journal.pmed.1001841 (PMC4469461; doi:10.1371/journal.pmed.1001841)

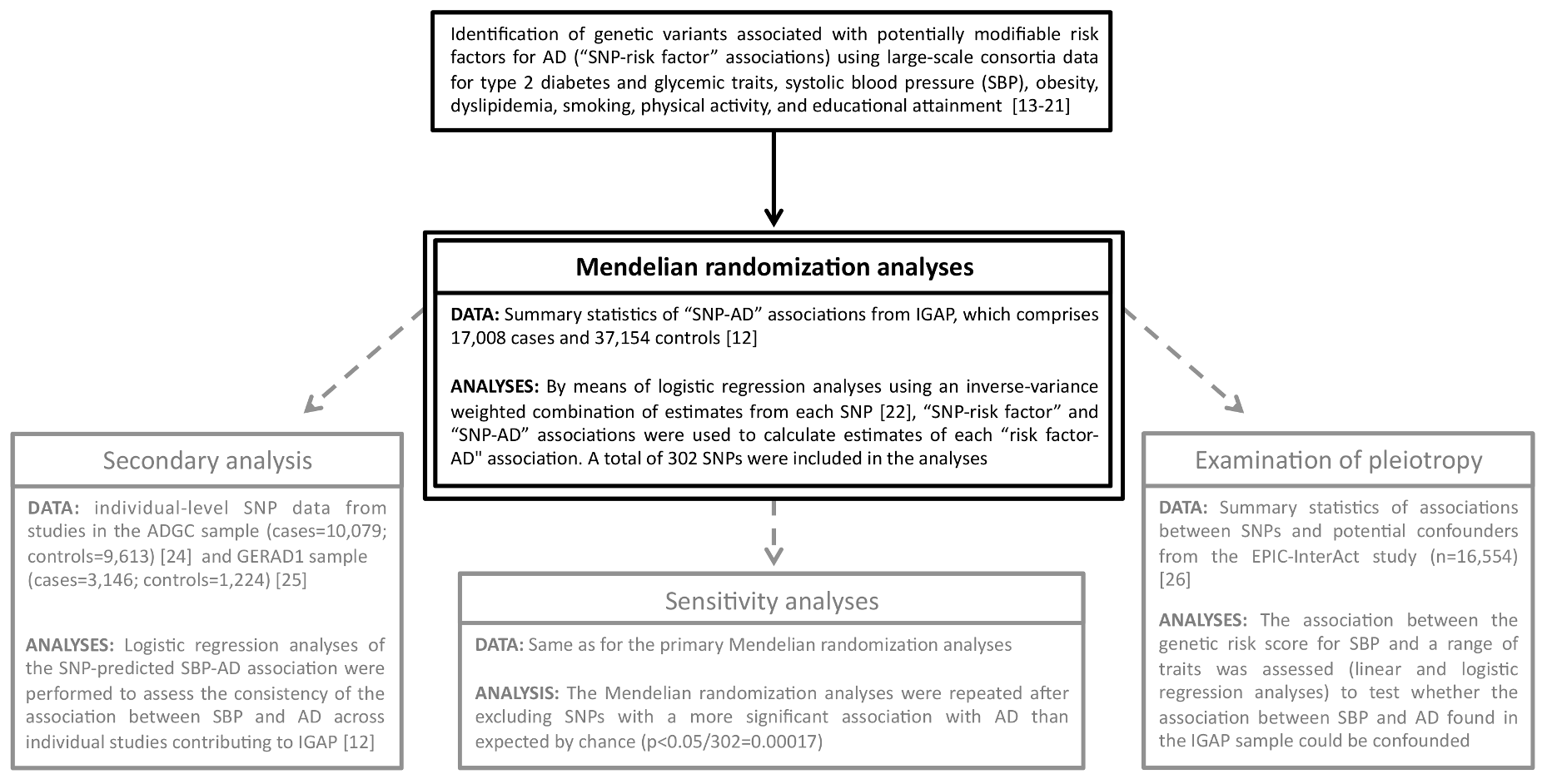

Supplement: S1 Fig — (TIF) [file pmed.1001841.s002.tif]

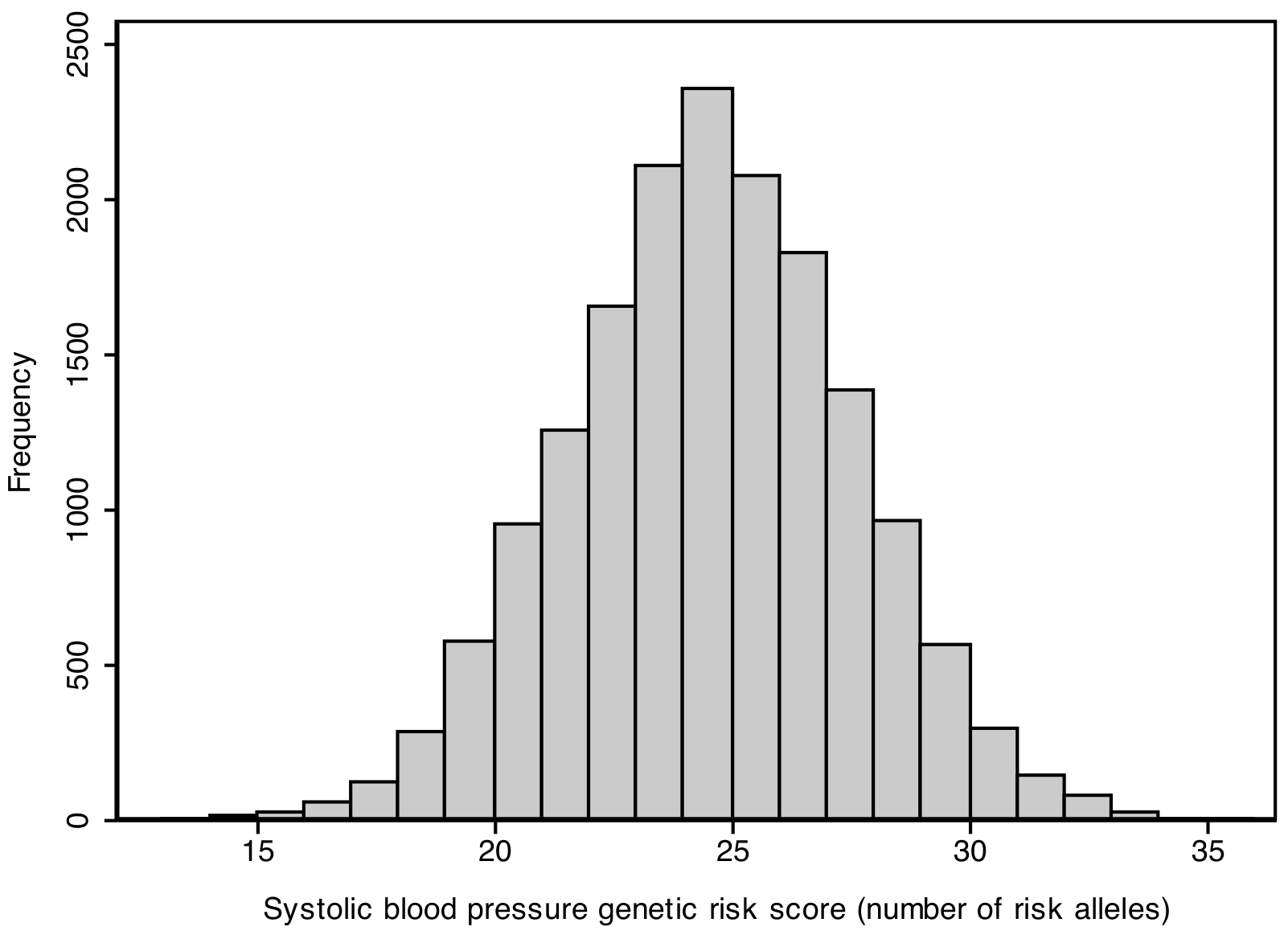

Supplement: S2 Fig — n = 16,691. (TIF) [file pmed.1001841.s003.tif]

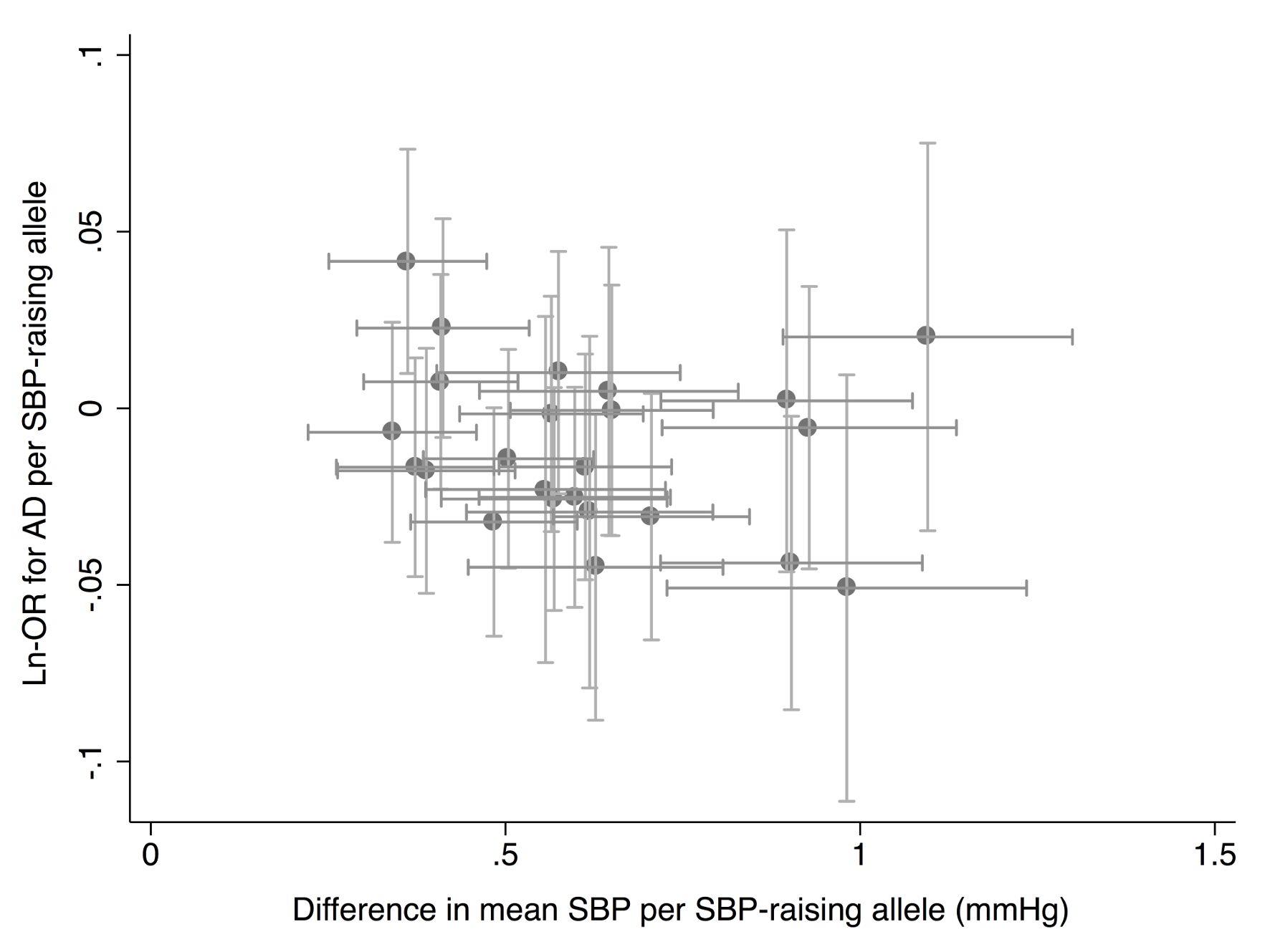

Supplement: S3 Fig — (TIF) [file pmed.1001841.s004.tif]

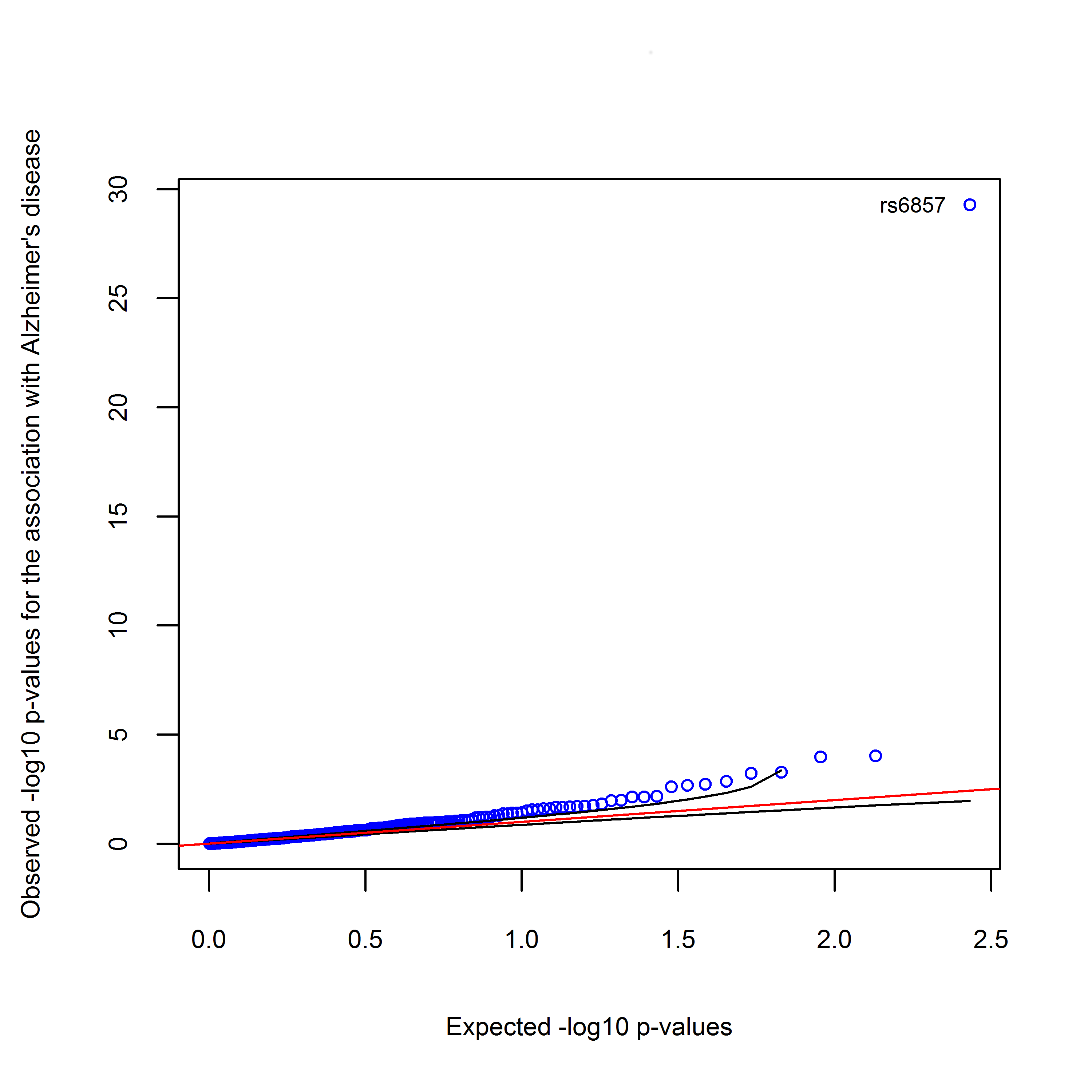

Supplement: S4 Fig — SNPs from all scores (N unique = 302) were LD-pruned and duplicates were removed to leave the 269 variants shown here. The SNP near APOE (rs6857) had a p-value of 2.5 × 10−575, which was truncated to 10−30 for display on the figure. (TIF) [file pmed.1001841.s005.tif]

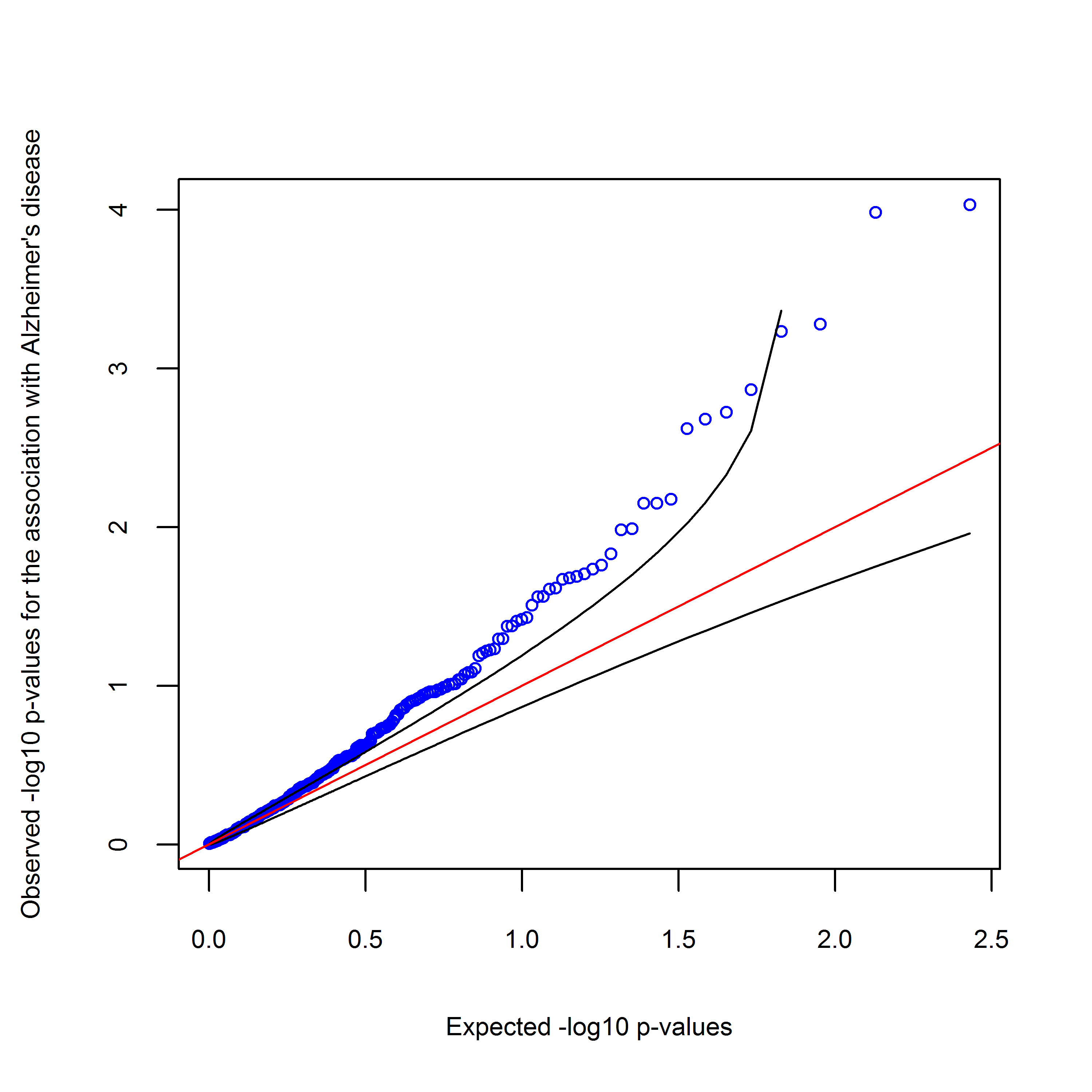

Supplement: S5 Fig — SNPs from all scores (N unique = 302) were LD-pruned and duplicates were removed to leave 269 variants. For the present plot, the SNP near APOE (rs6857, p = 2.5 × 10−575) was excluded (see S4 Fig). (TIF) [file pmed.1001841.s006.tif]

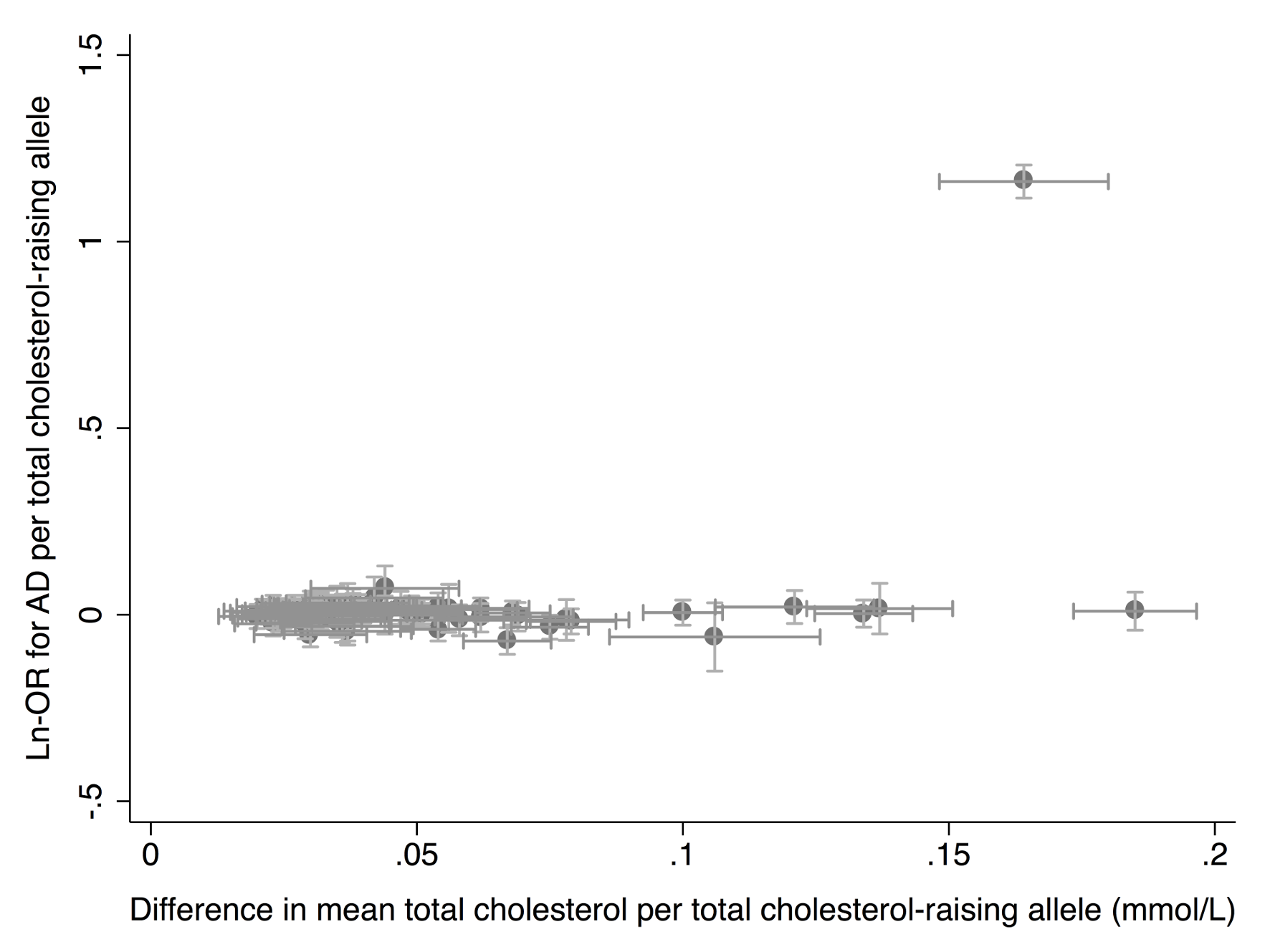

Supplement: S6 Fig — (TIF) [file pmed.1001841.s007.tif]

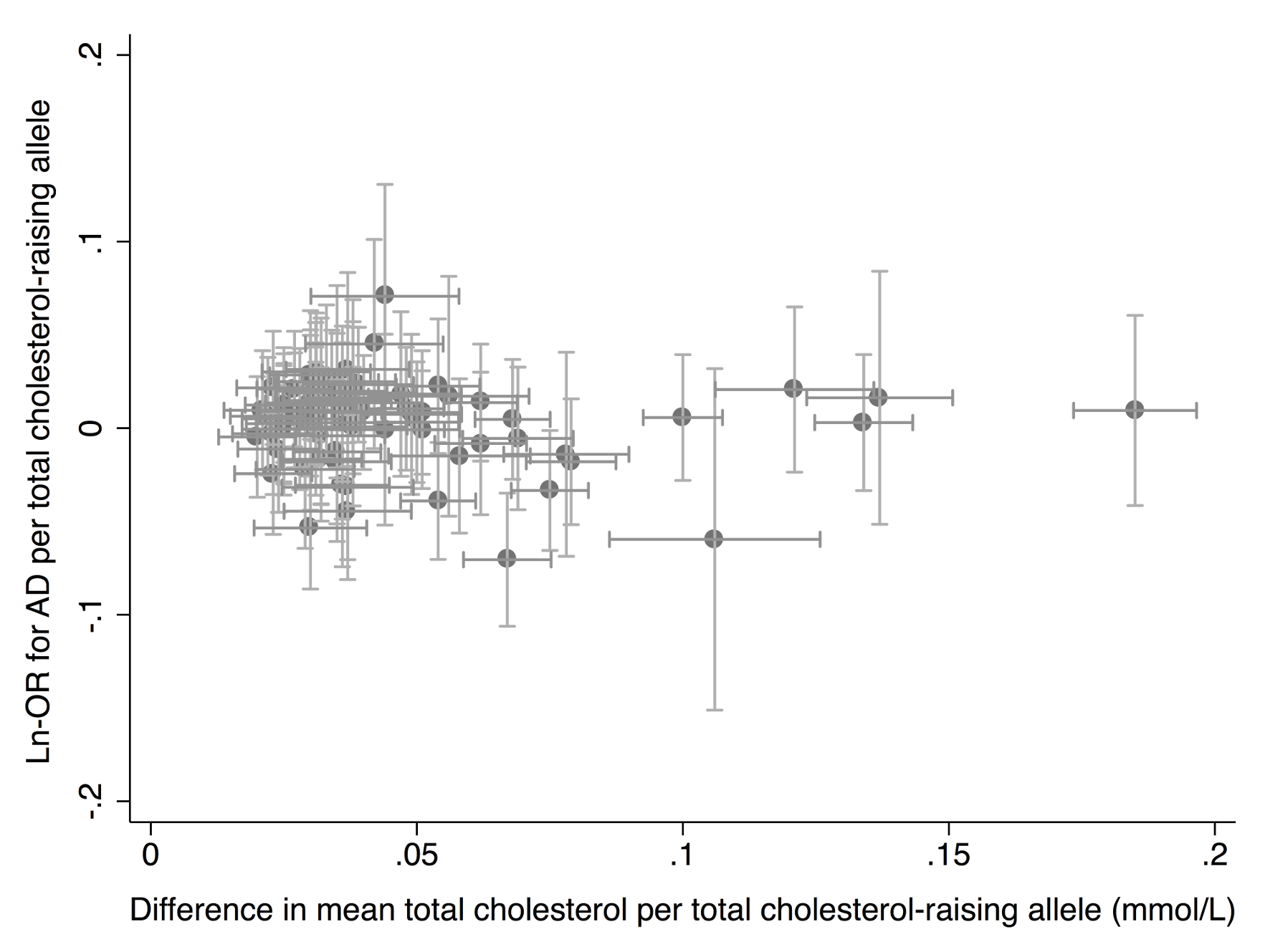

Supplement: S7 Fig — (TIF) [file pmed.1001841.s008.tif]

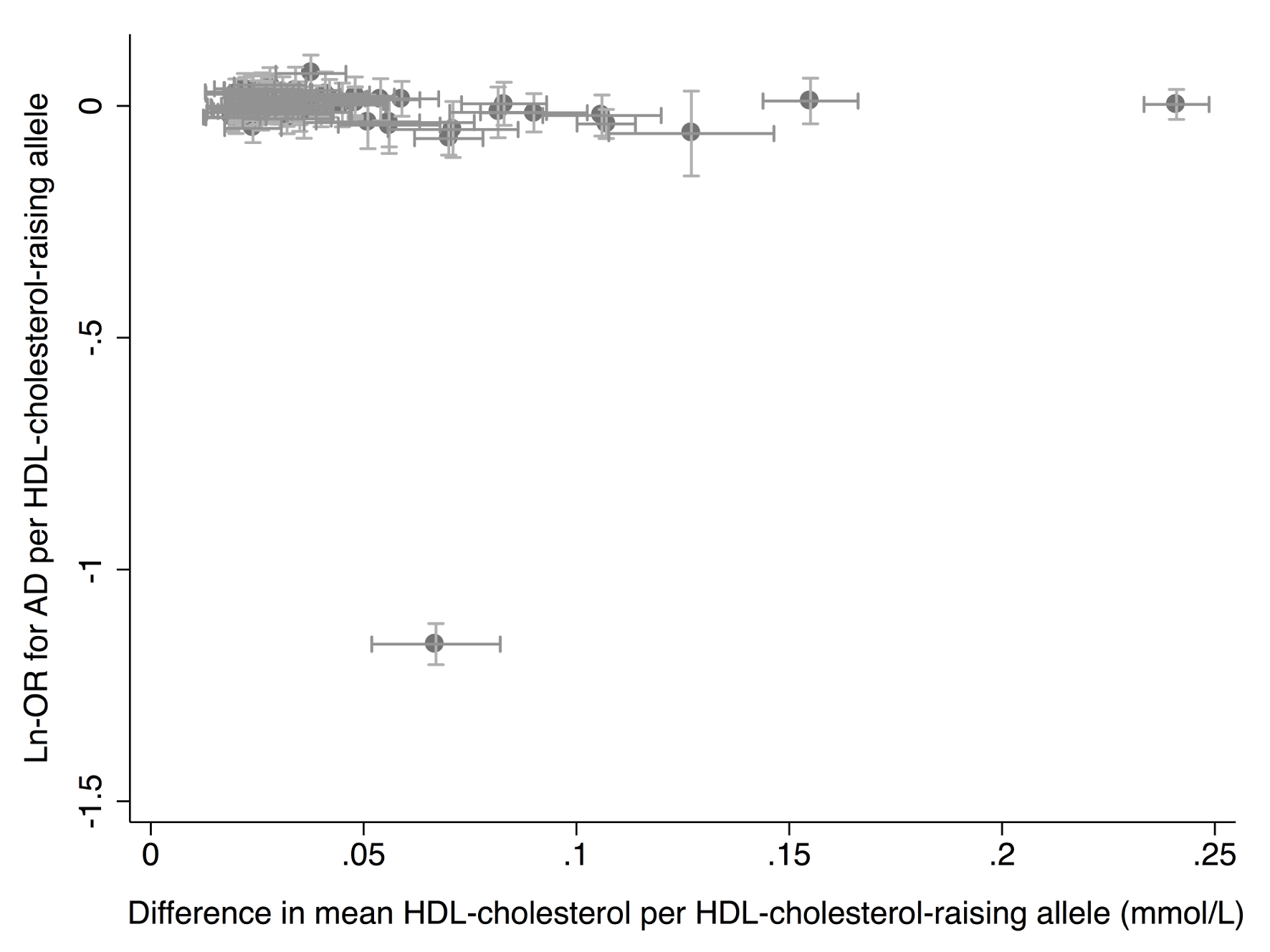

Supplement: S8 Fig — (TIF) [file pmed.1001841.s009.tif]

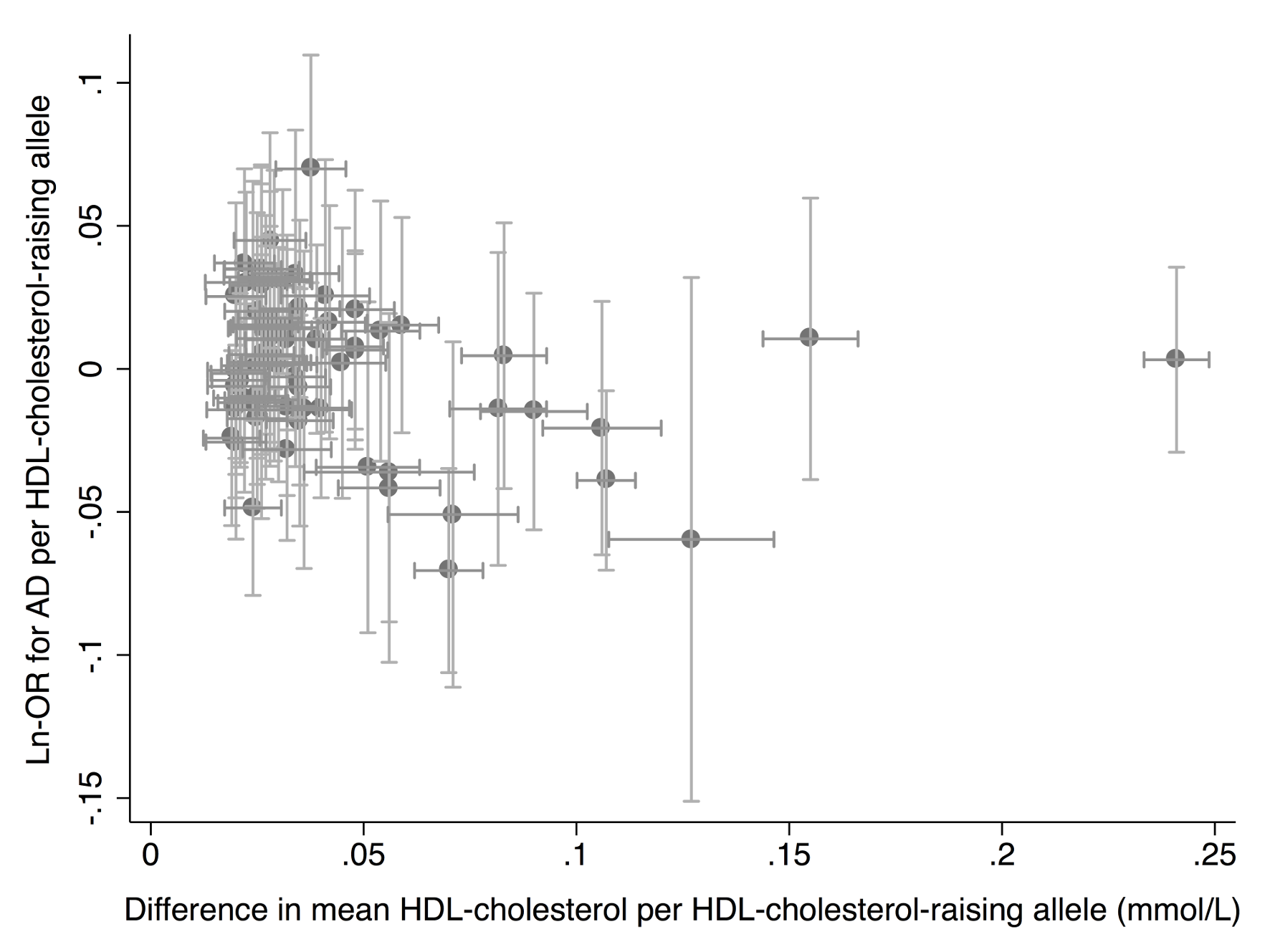

Supplement: S9 Fig — (TIF) [file pmed.1001841.s010.tif]

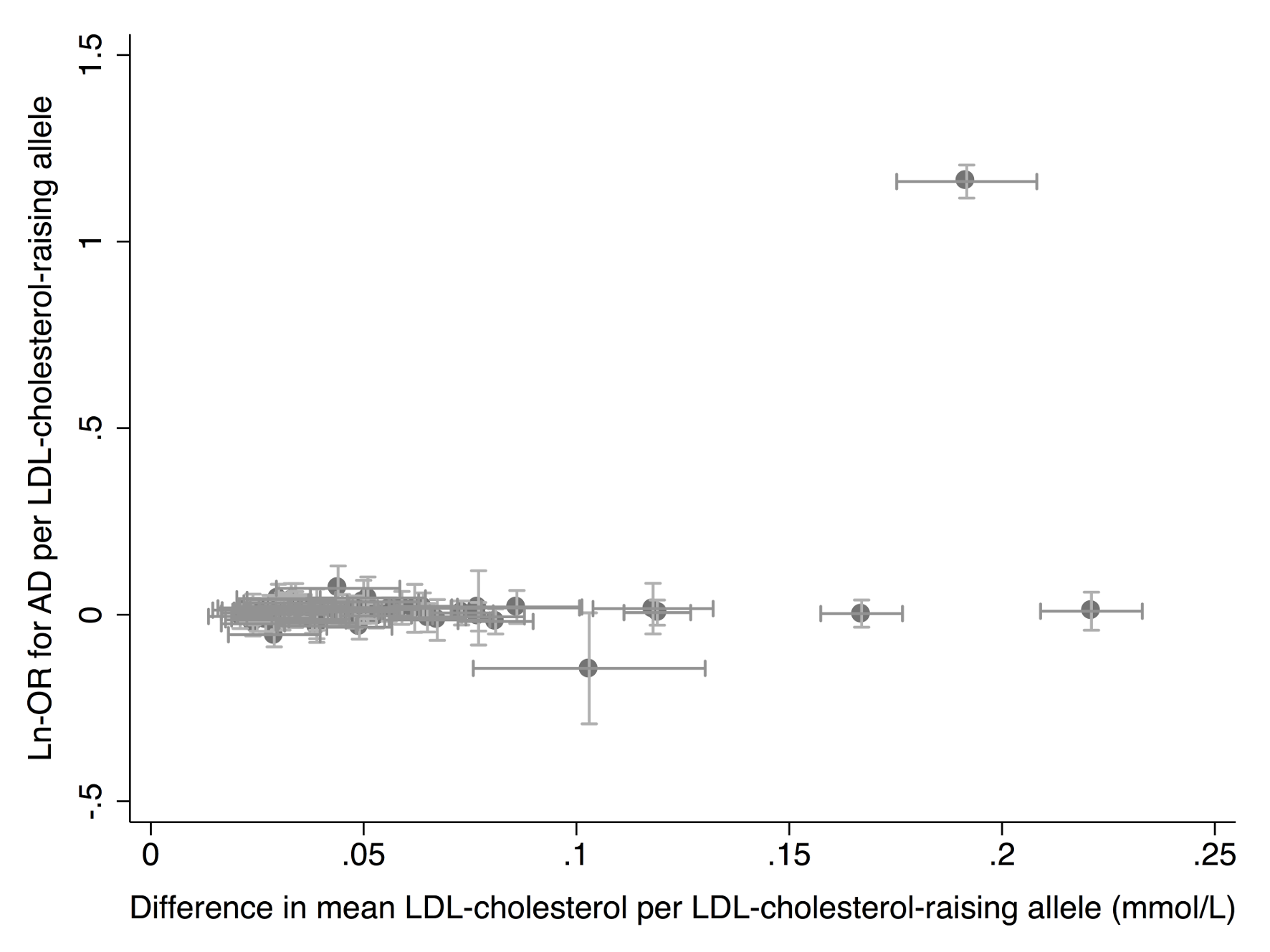

Supplement: S10 Fig — (TIF) [file pmed.1001841.s011.tif]

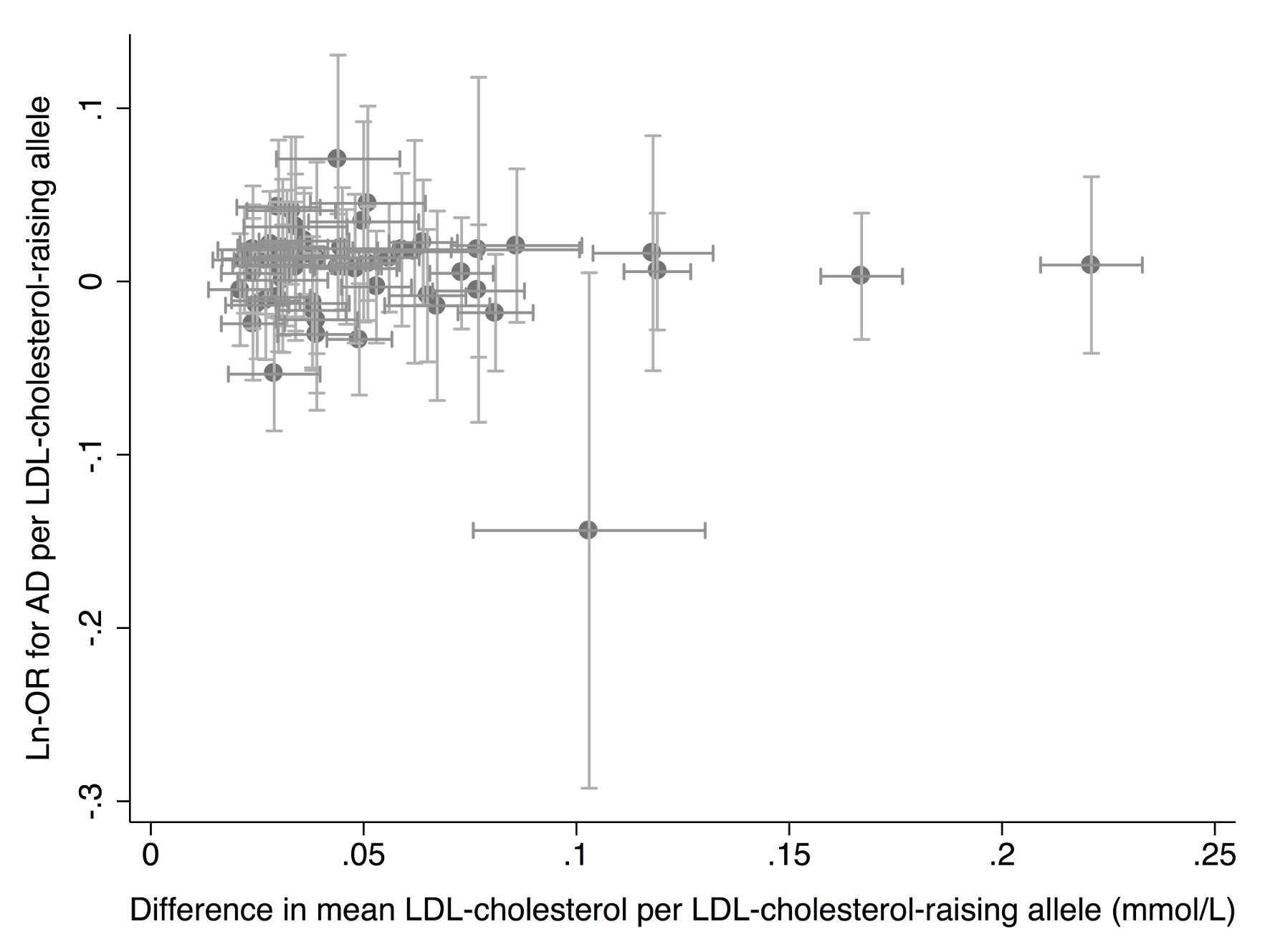

Supplement: S11 Fig — (TIF) [file pmed.1001841.s012.tif]

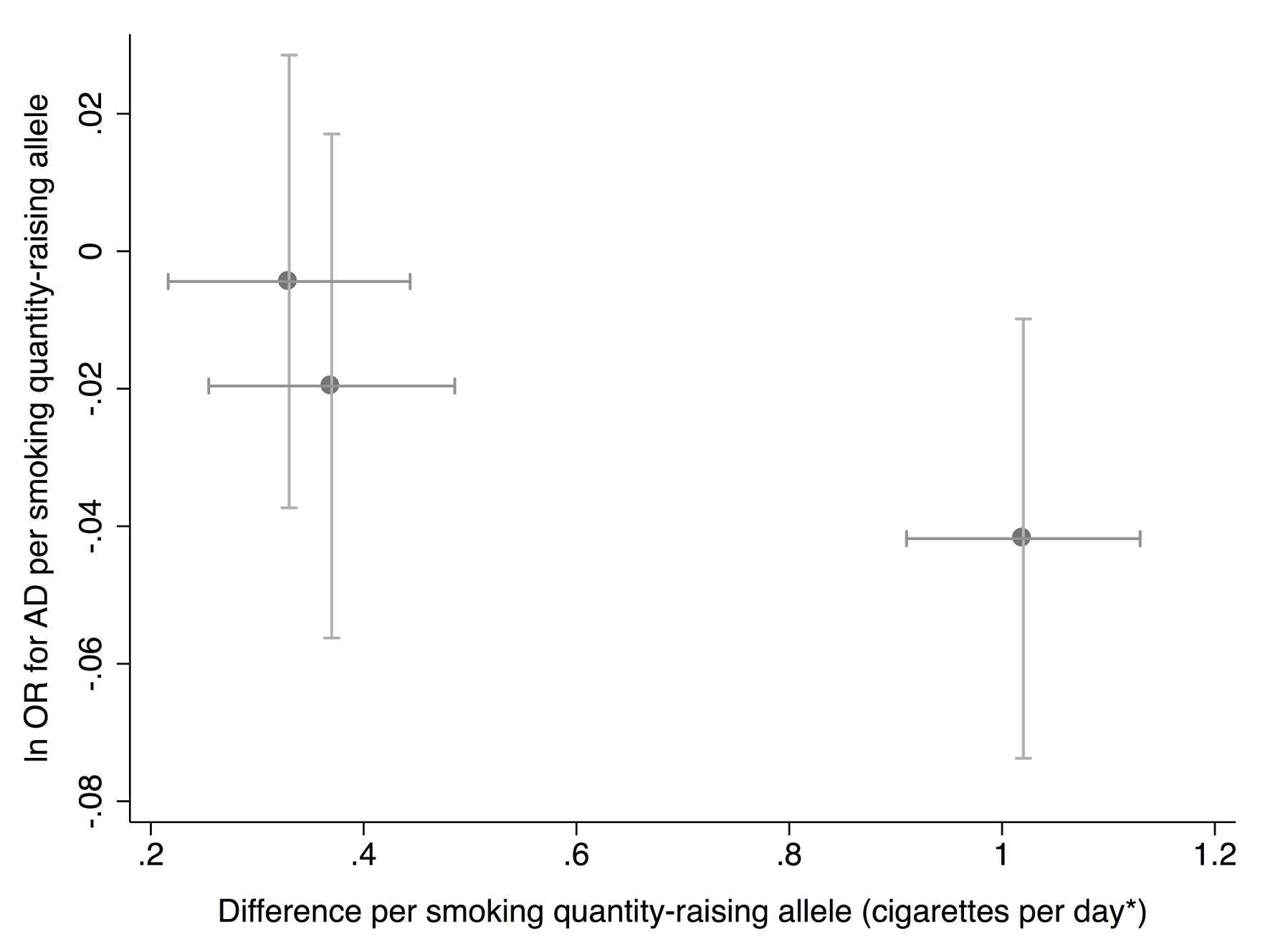

Supplement: S12 Fig — *Note that the effect sizes for the effects of SNPs on smoking quantity were estimated in current smokers only, while the associations with AD are not stratified by smoking status. (TIF) [file pmed.1001841.s013.tif]
