# Supplementary material for: Associations between Potentially Modifiable Risk Factors and Alzheimer Disease: A Mendelian Randomization Study
Source: PLoS Med. 2015 Jun 16;12(6):e1001841. doi: 10.1371/journal.pmed.1001841 (PMC4469461; doi:10.1371/journal.pmed.1001841)
Supplement: S1 Text — (DOCX) [file pmed.1001841.s015.docx]

**Supporting Text**

**Supplemental Methods**

**Alzheimer’s disease genetic data:** Summary statistics of SNP-AD associations were derived from the International Genomics of Alzheimer's Project (IGAP) and individual-level SNP data were derived from the Genetic and Environmental Risk in Alzheimer’s disease (GERAD1) Consortium and the Alzheimer’s Disease Genetics Consortium (ADGC). These data are described in further detail below:

*IGAP:* The International Genomics of Alzheimer's Project (IGAP) [1] is a large two-stage study based upon genome-wide association studies (GWAS) of AD from individuals of European ancestry. In stage 1, IGAP used genotyped and imputed data on 7055881 SNPs to meta-analyse four previously-published GWAS datasets consisting of 17008 AD cases and 37154 controls. The four GWAS datasets are: the European AD Initiative – EADI, the AD Genetics Consortium – ADGC, the Cohorts for Heart and Aging Research in Genomic Epidemiology consortium – CHARGE, and the Genetic and Environmental Risk in AD consortium – GERAD. Information regarding recruitment and diagnostic assessment is provided in detail elsewhere [1].

*GERAD imputed genotype data:* Data used in the preparation of this article were obtained from the Genetic and Environmental Risk for Alzheimer’s disease (GERAD) Consortium. The imputed GERAD sample comprised 3,177 AD cases and 7,277 controls with available age and gender data. Cases and elderly screened controls were recruited by the Medical Research Council (MRC) Genetic Resource for AD (Cardiff University; Institute of Psychiatry, London; Cambridge University; Trinity College Dublin), the Alzheimer’s Research UK(ARUK) Collaboration (University of Nottingham; University of Manchester; University of Southampton; University of Bristol; Queen’s University Belfast; the Oxford Project to Investigate Memory and Ageing (OPTIMA), Oxford University); Washington University, St Louis, United States; MRC PRION Unit, University College London; London and the South East Region AD project (LASER-AD), University College London; Competence Network of Dementia (CND) and Department of Psychiatry, University of Bonn, Germany; the National Institute of Mental Health (NIMH)AD Genetics Initiative. 6,129 population controls were drawn from large existing cohorts with available GWAS data, including the 1958 British Birth Cohort (1958BC) (http://www.b58cgene.sgul.ac.uk), the KORA F4 Study and the Heinz Nixdorf Recall Study. All AD cases met criteria for either probable (NINCDS-ADRDA, DSM-IV) or definite (CERAD) AD. All elderly controls were screened for dementia using the MMSE or ADAS-cog, were determined to be free from dementia at neuropathological examination or had a Braak score of 2.5 or lower. Genotypes from all cases and 4,617 controls were previously included in the AD GWAS by Harold and colleagues (2009). Genotypes for the remaining 2,660 population controls were obtained from WTCCC2. Imputation of the dataset was performed using IMPUTE2 and the 1000 genomes (http://www.1000genomes.org/) Dec2010 reference panel (NCBI build 37.1). The imputed data was then analysed using logistic regression including covariates for country of origin, gender, age and 3 principal components obtained with EIGENSTRAT software based on individual genotypes for the GERAD study participants.

*ADGC sample:* The final ADGC dataset comprises individuals from i.) Adult Changes in Thought (ACT)/ Electronic Medical Records and Genetics (eMERGE); ii.) National Institute on Aging (NIA) AD Centers (ADCs); iii.) AD Neuroimaging Initiative (ADNI); iv.) Multi-Site Collaborative Study for Genotype-Phenotype Associations in AD (GenADA) Study; v.) University of Miami/Vanderbilt University/Mt. Sinai School of Medicine (UM/VU/MSSM); vi.) Multi-Institutional Research in Alzheimer’s Genetic Epidemiology (MIRAGE) Study; vii.) Oregon Health and Science University (OHSU); viii.) NIA Late Onset AD (NIA-LOAD) Study; ix.) Translational Genomics Research Institute series 2 (TGEN2); x.) the Mayo Clinic; xi.) Rush University Religious Orders Study/Memory and Aging Project (ROS-MAP); xii.) University of Pittsburgh (UP); and xiii.) Washington University (WASHU). The covariate dataset included age, sex, autopsy status and APOE genotype. Age of onset, age at ascertainment, age at diagnosis or a combination of both age at ascertainment and age at death were available from each of the 15 cohorts. As in Naj et al. [2], for subjects with autopsy-confirmed diagnosis and no clinical diagnosis, the age at death was used for age at diagnosis. For all studies, the age used for CNEs was the age of last exam or age at death for those for whom age at exam was not available. Case and CNE subjects with age at symptom onset or age at death less than 60 were excluded from the analysis. We restricted our association analyses to individuals of European ancestry because there were insufficient subjects from non–European-ancestry groups to obtain meaningful results. More detailed information can be found in Naj et al [2]. Genotypes were from either Illumina or Affymetrix high-density SNP microarray. Each of the 15 raw datasets went through extensive sample and genotype quality control [2] before being imputed to Hapmap2 using MACH software with HapMap phase 2 (release 22) CEPH Utah pedigree (CEU) reference haplotypes and genotype data passing quality control as inference. Imputation quality was evaluated using the R2 statistics and only SNPs imputed with R2 ≥ 0⋅50 were included in the dataset. We obtained these quality-controlled Hapmap2 imputed datasets from the ADGC and performed further quality control both on samples and genotype data. Before merging the 15 datasets together, we checked each of the 14 datasets for the same genomic physical positions based on the ACT dataset which we used as the reference set. There is a known issue with strand ambiguous SNPs (A/T; C/G) and the Illumina microarray platform [3]. We took a conservative approach and removed strand ambiguous SNPs from each of the datasets. After merging the datasets, the combined dataset was further cleaned by applying minimum call rate of 98% and minimum minor allele frequency of 0⋅01. SNPs not in Hardy-Weinberg equilibrium (P < 10−6) were excluded from analysis. All these steps were performed using PLINK [4]. Two of the studies, NIA-LOAD and MIRAGE, were family based. We were interested in creating an ‘unrelated’ dataset and hence extracted an individual at random from each of the families from each of these studies. We also excluded individuals with missing case-control status and ended up with a dataset of 20394 individuals. To look at a) cryptic relatedness across studies and b) calculate principal components to account for population-specific variations in allele distribution on the SNPs, we considered a dataset with observed (i.e. not imputed) non strand ambiguous SNPs common across the 15 studies (no. of SNPs=21880). After filtering SNPs with pairwise LD (r2) < 0⋅20, we ended up with 17054 SNPs. We used both PLINK and KING-ROBUST for relatedness analysis. Our sample was quite heterogeneous and PLINK was unable to clearly separate the 2nd and 3rd degree relatives. On the other hand, KING-ROBUST provided unbiased (or approximately unbiased) kinship coefficient estimates for related individuals in this setting. Our final ‘unrelated’ dataset had a sample size was 19692 after excluding up to 3rd degree relatives (kinship >= 0⋅0442).

**Mendelian randomization analyses:** Given the association of the blood pressure score with AD and previous suggestions of different effects of blood pressure on AD risk dependent on age, we sought to assess whether the SNPs used in the MR analysis of systolic blood pressure were predictive of systolic blood pressure across the adult age range. We calculated a genetic risk score (the sum of the number of risk alleles for each of the SNPs) and conducted an age-stratified analysis on participants in the EPIC-InterAct study [5]. Specifically, we stratified the InterAct cohort into three age groups: <50 years old, 50-60 years old, and >60 years old and used linear regression to model the association of the genetic score with systolic blood pressure, adjusted for age, sex, centre and subcohort status [5].

**QQ plot:** To compare the distribution of p-values for the association with AD with that expected under a null distribution, we prepared a QQ plot of the p-values for association with AD for SNPs included across all traits (Se supplementary figure 3a and 3b). Prior to generating the plot, we first removed duplicates and LD-pruned the list of 302 SNPs (without regard to p-value) to r^2^<0.01 within a 500k window using PLINK [4]. This pruned the list to 269 variants.

**Supplemental Results**

**Abbreviations for individual study names in Fig. 1:** The Alzheimer's Disease Genetics Consortium (ADGC) comprises individuals from the Adult Changes in Thought (ACT)/ Electronic Medical Records and Genetics (eMERGE) study, the National Institute on Aging (NIA), Alzheimer Disease Centers (ADCs), the Alzheimer Disease Neuroimaging Initiative (ADNI) Study, the Multi-Site Collaborative Study for Genotype-Phenotype Associations in Alzheimers Disease (GenADA) Study, the University of Miami/Vanderbilt University/Mt. Sinai School of Medicine (UM/VU/MSSM), the Multi-Institutional Research in Alzheimer's Genetic Epidemiology (MIRAGE) Study, Oregon Health and Science University (OHSU), the National Institute of Aging-Late-onset Alzheimer's Disease (NIA-LOAD) Study, the Translational Genomics Research Institute series 2 (TGEN2), the Mayo Clinic (MAYO), the Rush University Religious Orders Study/Memory and Aging Project (ROSMAP), the University of Pittsburgh (UPITT), and Washington University (WU). Genetic and Environmental Risk in Alzheimer's Disease (GERAD). International Genomics of Alzheimer's Project (IGAP).

**Acknowledgments**

We thank the International Genomics of Alzheimer's Project (IGAP) for providing summary results data for these analyses. The investigators within IGAP contributed to the design and implementation of IGAP and/or provided data but did not participate in analysis or writing of this report. IGAP was made possible by the generous participation of the control subjects, the patients, and their families. The i–Select chips were funded by the French National Foundation on Alzheimer's disease and related disorders. EADI was supported by the LABEX (laboratory of excellence program investment for the future) DISTALZ grant, Inserm, Institut Pasteur de Lille, Université de Lille 2 and the Lille University Hospital. GERAD was supported by the Medical Research Council (Grant n° 503480), Alzheimer's Research UK (Grant n° 503176), the Wellcome Trust (Grant n° 082604/2/07/Z) and German Federal Ministry of Education and Research (BMBF): Competence Network Dementia (CND) grant n° 01GI0102, 01GI0711, 01GI0420. CHARGE was partly supported by the NIH/NIA grant R01 AG033193 and the NIA AG081220 and AGES contract N01–AG–12100, the NHLBI grant R01 HL105756, the Icelandic Heart Association, and the Erasmus Medical Center and Erasmus University. ADGC was supported by the NIH/NIA grants: U01 AG032984, U24 AG021886, U01 AG016976, and the Alzheimer's Association grant ADGC–10–196728.

We also thank the Alzheimer’s Disease Genetics Consortium (ADGC) investigators: Marilyn S. Albert^1^, Roger L. Albin^2-4^, Liana G. Apostolova^5^, Steven E. Arnold^6^, Sanjay Asthana^7-9^,Craig S. Atwood^9,7^, Clinton T. Baldwin^10^, Robert C. Barber^11^, Michael M. Barmada^12^, Lisa L. Barnes^13,14^, Thomas G. Beach^15^, James T. Becker^16^, Gary W. Beecham^17,18^, Duane Beekly^19^,Eileen H. Bigio^20,21^, Thomas D. Bird^22,23^, Deborah Blacker^24,25^, Bradley F. Boeve^26^, James D. Bowen^27^, Adam Boxer^28^, James R. Burke^29^, Joseph D. Buxbaum^30-32^, Nigel J. Cairns^33^, Laura B. Cantwell^34^, Chuanhai Cao^35^, Chris S. Carlson^36^, Cynthia M. Carlsson^8^, Regina M. Carney^37^, Minerva M. Carrasquillo^38^, Steven L. Carroll^39^, Helena C. Chui^40^, David G. Clark^41^, Jason Corneveaux^42^, David H. Cribbs^44^, Elizabeth A. Crocco^37^, Carlos Cruchaga^45^, Philip L. De Jager^46,47^, Charles DeCarli^48^, F. Yesim Demirci^12^, Malcolm Dick^49^, Dennis W. Dickson^38^, Ranjan Duara^50^,Nilufer Ertekin-Taner^38,51^, Denis Evans^52^, Kelley M. Faber^53^, Kenneth B. Fallon^39^, Martin R. Farlow^59^, Lindsay A. Farrer^54-58^, Steven Ferris^60^, Tatiana M. Foroud^53^, Matthew P. Frosch^61^,Douglas R. Galasko^62^, Marla Gearing^63,64^, Daniel H. Geschwind^65^, Bernardino Ghetti^66^, John R.Gilbert^17,18^, Jonathan D. Glass^67^, Alison M. Goate^45^, Neill R. Graff-Radford^38,51^, Robert C. Green^68^, John H. Growdon^69^, Jonathan L. Haines^70^, Hakon Hakonarson^71^, Ronald L.Hamilton^72^, Kara L. Hamilton-Nelson^17^, John Hardy^73^, Lindy E. Harrell^41^, Elizabeth Head^74^, Lawrence S. Honig^75^, Ryan M. Huebinger^76^, Matthew J. Huentelman^42^, Christine M. Hulette^77^, Bradley T. Hyman^69^, Gail P. Jarvik^78,79^, Gregory A. Jicha^80^, Lee-Way Jin^81^, Gyungah Jun^10,54,58^, M. Ilyas Kamboh^12,82^, Anna Karydas^28^, Jeffrey A. Kaye^83,84^, Ronald Kim^85^, Neil W. Kowall^57,86^, Joel H. Kramer^87^, Walter A. Kukull^88^, Brian W. Kunkle^17^, Frank M. LaFerla^89^, James J. Lah^67^, James B.Leverenz^90^, Allan I. Levey^67^, Ge Li^91^, Andrew P. Lieberman^92^, Chiao-Feng Lin^34^, Oscar L. Lopez^82^,Kathryn L. Lunetta^54^, Constantine G. Lyketsos^93^, Wendy J. Mack^94^, Daniel C. Marson^41^, Eden R.Martin^17,18^, Frank Martiniuk^95^, Deborah C. Mash^96^, Eliezer Masliah^62,97^, Richard Mayeux^75,98,99^, Wayne C. McCormick^43^, Susan M. McCurry^100^, Andrew N. McDavid^36^, Ann C. McKee^57,86^, Marsel Mesulam^20,101^, Bruce L. Miller^28^, Carol A. Miller^102^, Joshua W. Miller^81^, Thomas J. Montine^103^, John C. Morris^33,104^, Jill R. Murrell^53,66^, Amanda J. Myers^37^, Adam C. Naj^34^, John M. Olichney^48^, Vernon S. Pankratz^105^, Joseph E. Parisi^106^, Amanda Partch^34^, Henry L. Paulson^107^, Margaret A. Pericak-Vance^17,18^, William Perry^17^, Elaine Peskind^91^, Ronald C. Petersen^26^, Aimee Pierce^44^, Wayne W. Poon^49^, Huntington Potter^108^, Joseph F. Quinn^83^, Ashok Raj^35^, Murray Raskind^91^, Eric M. Reiman^42,109-111^, Barry Reisberg^60,112^, Christiane Reitz^75,98,99^, John M. Ringman^5^, Erik D. Roberson^41^, Ekaterina Rogaeva^113^, Howard J. Rosen^28^, Roger N. Rosenberg^114^, Mark A. Sager^8^,Mary Sano^31^, Gerard D. Schellenberg^34^, Julie A. Schneider^13,115^, Lon S. Schneider^40,116^, William W. Seeley^28^, Amanda G. Smith^35^, Joshua A. Sonnen^103^, Salvatore Spina^66^, Peter St George-Hyslop^113,117^, Robert A. Stern^57^, Rudolph E. Tanzi^69^, Tricia A. Thornton-Wells^118^, John Q. Trojanowski^34^, Juan C. Troncoso^119^, Debby W. Tsuang^23,91^, Otto Valladares^34^, Vivianna M. VanDeerlin^34^, Linda J. Van Eldik^120^, Badri N. Vardarajan^75,98,99^, Harry V. Vinters^5,121^, Jean Paul Vonsattel^122^, Li-San Wang^34^, Sandra Weintraub^20,123^, Kathleen A. Welsh-Bohmer^29,124^, Jennifer Williamson^75^, Sarah Wishnek^17^, Randall L. Woltjer^125^, Clinton B. Wright^126^, Steven G. Younkin^38^,Chang-En Yu^43^, Lei Yu^13^.

ADGC affiliations: 1. Department of Neurology, Johns Hopkins University, Baltimore, Maryland, 2. Department of Neurology, University of Michigan, Ann Arbor, Michigan, 3. Geriatric Research, Education and Clinical Center (GRECC), VA Ann Arbor Healthcare System (VAAAHS), Ann Arbor, Michigan, 4. Michigan Alzheimer Disease Center, Ann Arbor, Michigan, 5. Department of Neurology, University of California Los Angeles, Los Angeles, California, 6. Department of Psychiatry, University of Pennsylvania Perelman School of Medicine, Philadelphia, Pennsylvania, 7. Geriatric Research, Education and Clinical Center (GRECC), University of Wisconsin, Madison, Wisconsin, 8. Department of Medicine, University of Wisconsin, Madison, Wisconsin, 9. Wisconsin Alzheimer's Institute, Madison, Wisconsin, 10. Department of Medicine (Genetics Program), Boston University, Boston, Massachusetts, 11. Department of Pharmacology and Neuroscience, University of North Texas Health Science Center, Fort Worth, Texas, 12. Department of Human Genetics, University of Pittsburgh, Pittsburgh, Pennsylvania, 13. Department of Neurological Sciences, Rush University Medical Center, Chicago, Illinois, 14. Department of Behavioral Sciences, Rush University Medical Center, Chicago, Illinois, 15. Civin Laboratory for Neuropathology, Banner Sun Health Research Institute, Phoenix, Arizona, 16. Departments of Psychiatry, Neurology, and Psychology, University of Pittsburgh School of Medicine, Pittsburgh,Pennsylvania, 17. The John P. Hussman Institute for Human Genomics, University of Miami, Miami, Florida, 18. Dr. John T. Macdonald Foundation Department of Human Genetics, University of Miami, Miami, Florida, 19. NationalAlzheimer's Coordinating Center, University of Washington, Seattle, Washington, 20. Cognitive Neurology and Alzheimer's Disease Center, Northwestern University Feinberg School of Medicine, Chicago, Illinois, 21. Department of Pathology, Northwestern University Feinberg School of Medicine, Chicago, Illinois, 22. Department of Neurology, University of Washington, Seattle, Washington, 23. VA Puget Sound Health Care System/GRECC, Seattle, Washington, 24. Department of Epidemiology, Harvard School of Public Health, Boston, Massachusetts, 25. Department of Psychiatry, Massachusetts General Hospital/Harvard Medical School, Boston, Massachusetts, 26. Department of Neurology, Mayo Clinic, Rochester, Minnesota, 27. Swedish Medical Center, Seattle, Washington, 28. Department of Neurology, University of California San Francisco, San Francisco, California, 29. Department of Medicine, Duke University, Durham, North Carolina, 30. Department of Neuroscience, Mount Sinai School of Medicine, New York, New York, 31. Department of Psychiatry, Mount Sinai School of Medicine, New York, New York, 32. Departments of Genetics and Genomic Sciences, Mount Sinai School of Medicine, New York, New York, 33. Department of Pathology and Immunology, Washington University, St. Louis, Missouri, 34. Department of Pathology and Laboratory Medicine, University of Pennsylvania Perelman School of Medicine, Philadelphia, Pennsylvania, 35. USF Health Byrd Alzheimer's Institute, University of South Florida, Tampa, Florida, 36. Fred Hutchinson Cancer Research Center, Seattle, Washington, 37. Department of Psychiatry and Behavioral Sciences, Miller School of Medicine, University of Miami, Miami, Florida, 38. Department of Neuroscience, Mayo Clinic, Jacksonville, Florida, 39. Department of Pathology, University of Alabama at Birmingham, Birmingham, Alabama, 40. Department of Neurology, University of Southern California, Los Angeles, California, 41. Department of Neurology, University of Alabama at Birmingham, Birmingham, Alabama, 42. Neurogenomics Division, Translational Genomics Research Institute, Phoenix, Arizona, 43. Department of Medicine, University of Washington, Seattle, Washington, 44. Department of Neurology, University of California Irvine, Irvine, California, 45. Department of Psychiatry and Hope Center Program on Protein Aggregation and Neurodegeneration, Washington University School of Medicine, St. Louis, Missouri, 46. Program in Translational Neuro Psychiatric Genomics, Institute for the Neurosciences, Department of Neurology & Psychiatry, Brigham and Women's Hospital and Harvard Medical School, Boston, Massachusetts, 47. Program in Medical and Population Genetics, Broad Institute, Cambridge, Massachusetts, 48. Department of Neurology, University of California Davis, Sacramento, California, 49. Institute for Memory Impairments and Neurological Disorders, University of California Irvine, Irvine, California, 50. Wien Center for Alzheimer's Disease and Memory Disorders, Mount Sinai Medical Center, Miami Beach, Florida, 51. Department of Neurology, Mayo Clinic, Jacksonville, Florida, 52. Rush Institute for Healthy Aging, Department of Internal Medicine, Rush University Medical Center, Chicago, Illinois, 53. Department of Medical and Molecular Genetics, Indiana University, Indianapolis, Indiana, 54. Department of Biostatistics, Boston University, Boston, Massachusetts, 55. Department of Epidemiology, Boston University, Boston, Massachusetts, 56. Department of Medicine (Biomedical Genetics), Boston University, Boston, Massachusetts, 57. Department of Neurology, Boston University, Boston, Massachusetts, 58. Department of Ophthalmology, Boston University, Boston, Massachusetts, 59. Department of Neurology, Indiana University, Indianapolis, Indiana, 60. Department of Psychiatry, New York University, New York, New York, 61. C.S. Kubik Laboratory for Neuropathology, Massachusetts General Hospital, Charlestown, Massachusetts, 62. Department of Neurosciences, University of California San Diego, La Jolla, California, 63. Department of Pathology and Laboratory Medicine, Emory University, Atlanta, Georgia, 64. Emory Alzheimer's Disease Center, Emory University, Atlanta, Georgia, 65. Neurogenetics Program, University of California Los Angeles, Los Angeles, California, 66. Department of Pathology and Laboratory Medicine, Indiana University, Indianapolis, Indiana, 67. Department of Neurology, Emory University, Atlanta, Georgia, 68. Division of Genetics, Department of Medicine and Partners Center for Personalized Genetic Medicine, Brigham and Women's Hospital and Harvard Medical School, Boston, Massachusetts, 69. Department of Neurology, Massachusetts General Hospital/Harvard Medical School, Boston, Massachusetts, 70. Department of Epidemiology and Biostatistics, Case Western Reserve University, Cleveland, Ohio, 71. Center for Applied Genomics, Children's Hospital of Philadelphia, Philadelphia, Pennsylvania, 72. Department of Pathology (Neuropathology), University of Pittsburgh, Pittsburgh, Pennsylvania, 73. Institute of Neurology, University College London, Queen Square, London, UK, 74. Sanders-Brown Center on Aging, Department of Molecular and Biomedical Pharmacology, University of Kentucky, Lexington, Kentucky, 75. Taub Institute on Alzheimer's Disease and the Aging Brain, Department of Neurology, Columbia University, New York, NewYork, 76. Department of Surgery, University of Texas Southwestern Medical Center, Dallas, Texas, 77. Department of Pathology, Duke University, Durham, North Carolina, 78. Department of Genome Sciences, University of Washington, Seattle, Washington, 79. Department of Medicine(Medical Genetics), University of Washington, Seattle, Washington, 80. Sanders-Brown Center on Aging, Department Neurology, University of Kentucky, Lexington, Kentucky, 81. Department of Pathology and Laboratory Medicine, University of California Davis, Sacramento, California, 82. University of Pittsburgh Alheimer's Disease Research Center, Pittsburgh, Pennsylvania, 83. Department of Neurology, Oregon Health & Science University, Portland, Oregon, 84. Department of Neurology, Portland Veterans Affairs Medical Center, Portland, Oregon, 85. Department of Pathology and Laboratory Medicine, University of California Irvine, Irvine, California, 86. Department of Pathology, Boston University, Boston, Massachusetts, 87. Department of Neuropsychology, University of California San Francisco, San Francisco, California, 88. Department of Epidemiology, University of Washington, Seattle, Washington, 89. Department of Neurobiology and Behavior, University of California Irvine, Irvine, California, 90. Cleveland Clinic Lou Ruvo Center for Brain Health, Cleveland Clinic, Cleveland, Ohio, 91. Department of Psychiatry and Behavioral Sciences, University of Washington School of Medicine, Seattle, Washington, 92. Department of Pathology, University of Michigan, Ann Arbor,Michigan, 93. Department of Psychiatry, Johns Hopkins University, Baltimore, Maryland, 94. Department of Preventive Medicine, University of Southern California, Los Angeles, California, 95. Department of Medicine-Pulmonary, New York University, New York, New York, 96. Department of Neurology, University of Miami, Miami, Florida, 97. Department of Pathology, University of California San Diego, La Jolla, California, 98. Department of Neurology, Columbia University, New York, New York, 99. Gertrude H. Sergievsky Center, Columbia University, New York, New York, 100. School of Nursing Northwest Research Group on Aging, University of Washington, Seattle, Washington, 101. Department of Neurology, Northwestern University Feinberg School of Medicine, Chicago, Illinois, 102. Department of Pathology, University of Southern California, Los Angeles, California, 103. Department of Pathology, University of Washington, Seattle, Washington, 104. Department of Neurology, Washington University, St. Louis, Missouri, 105. Department of Biostatistics, Mayo Clinic, Rochester, Minnesota, 106. Department of Laboratory Medicine and Pathology, Mayo Clinic, Rochester, Minnesota, 107. Michigan Alzheimer's Disease Center, Department of Neurology, University of Michigan, Ann Arbor, Michigan, 108. Department of Neurology, University of Colorado School of Medicine, Aurora, Colorado, 109. Arizona Alzheimer’s Consortium, Phoenix, Arizona, 110. Department of Psychiatry, University of Arizona, Phoenix, Arizona, 111. Banner Alzheimer's Institute, Phoenix,Arizona, 112. Alzheimer's Disease Center, New York University, New York, New York, 113. Tanz Centre for Research in Neurodegenerative Disease, University of Toronto, Toronto, Ontario, 114. Department of Neurology, University of Texas Southwestern, Dallas, Texas, 115. Department of Pathology (Neuropathology), Rush University Medical Center, Chicago, Illinois, 116. Departmentof Psychiatry, University of Southern California, Los Angeles, California, 117. Cambridge Institute for Medical Research and Department of Clinical Neurosciences, University of Cambridge, Cambridge, UK, 118. Center for Human Genetics and Research, Department of Molecular Physiology and Biophysics, Vanderbilt University, Nashville, Tennessee, 119. Department of Pathology, Johns Hopkins University, Baltimore, Maryland, 120. Sanders-Brown Center on Aging, Department of Anatomy and Neurobiology, University of Kentucky, Lexington, Kentucky, 121. Department of Pathology & Laboratory Medicine, University of California Los Angeles, Los Angeles, California, 122. Taub Institute on Alzheimer's Disease and the Aging Brain, Department of Pathology, Columbia University, New York, New York, 123. Department of Psychiatry, Northwestern University Feinberg School of Medicine, Chicago, Illinois, 124. Department of Psychiatry & Behavioral Sciences, Duke University, Durham, North Carolina, 125. Department of Pathology, Oregon Health & Science University, Portland, Oregon, 126. Evelyn F. McKnight Brain Institute, Department of Neurology, Miller School of Medicine, University of Miami, Miami, Florida.

We also thank the Genetic and Environmental Risk for Alzheimer's disease Consortium (GERAD1) investigators: Denise Harold^1^, Richard Abraham^1^, Paul Hollingworth^1^, Rebecca Sims^1^, Amy Gerrish^1^, Jade Chapman^1^, Giancarlo Russo^1^, Marian Hamshere^1^, Jaspreet Singh Pahwa^1^, Valentina Escott-Price^1^, Nandini Badarinarayan^1^, Kimberley Dowzell^1^, Amy Williams^1^, Nicola Jones^1^, Charlene Thomas^1^, Alexandra Stretton^1^, Angharad Morgan^1^, Sarah Taylor^1^, Simon Lovestone^2^, John Powell^2^, Petroula Proitsi^2^, Michelle K Lupton^2^, Carol Brayne^3^, David C. Rubinsztein^4^, Michael Gill^5^, Brian Lawlor^5^, Aoibhinn Lynch^5^, Kevin Morgan^6^, Kristelle Brown^6^, Peter Passmore^7^, David Craig^7^, Bernadette McGuinness^7^, Stephen Todd^7^, Janet Johnston^7^, Clive Holmes^8^, David Mann^9^, A. David Smith^10^, Seth Love^11^, Patrick G. Kehoe^11^, John Hardy^12^, Simon Mead^13^, Nick Fox^14^, Martin Rossor^14^, John Collinge^13^, Wolfgang Maier^15^, Frank Jessen^15^, Reiner Heun^15^, Britta Schürmann^15^, Alfredo Ramirez^15^, Tim Becker^44^_,_ Christine Herold^44^, André Lacour^44^, Dmitriy Drichel^44^, Hendrik van den Bussche^16^, Isabella Heuser^17^, Johannes Kornhuber^18^, Jens Wiltfang^19^, Martin Dichgans^20,21^, Lutz Frölich^22^, Harald Hampel^23,24^, Michael Hüll^25^, Dan Rujescu^24^, Alison Goate^27^, John S.K. Kauwe^28^, Carlos Cruchaga^27^, Petra Nowotny^27^, John C. Morris^26^, Kevin Mayo^26^, Gill Livingston^31^, Nicholas J. Bass^31^, Hugh Gurling^31^, Andrew McQuillin^32^, Rhian Gwilliam^33^, Panagiotis Deloukas^33^, Ammar Al-Chalabi^34^, Christopher E. Shaw^34^, Andrew B. Singleton^35^, Rita Guerreiro^35, 44^, Thomas W. Mühleisen^36,37^, Markus M. Nöthen^36,37,^ Susanne Moebus^38^, Karl-Heinz Jöckel^38^, Norman Klopp^39^, H-Erich Wichmann^39,40,41,^ Minerva M. Carrasquillo^42^, V. Shane Pankratz^43^, Steven G. Younkin^42^, Peter Holmans^1^, Michael ODonovan^1^, Michael J.Owen^1^, Julie Williams^1^.

GERAD affiliations: ^1^Medical Research Council (MRC) Centre for Neuropsychiatric Genetics and Genomics, Neurosciences and Mental Health Research Institute, Department of Psychological Medicine and Neurology, School of Medicine, Cardiff University, Cardiff, UK. ^2^Kings College London, Institute of Psychiatry, Department of Neuroscience, De Crespigny Park, Denmark Hill, London. ^3^Institute of Public Health, University of Cambridge, Cambridge, UK. ^4^Cambridge Institute for Medical Research, University of Cambridge, Cambridge, UK. ^5^Mercers Institute for Research on Aging, St. James Hospital and Trinity College, Dublin, Ireland. ^6^Institute of Genetics, Queens Medical Centre, University of Nottingham, NG7 2UH, UK. ^7^Ageing Group, Centre for Public Health, School of Medicine, Dentistry and Biomedical Sciences, Queens University Belfast, UK. ^8^Division of Clinical Neurosciences, School of Medicine, University of Southampton, Southampton, UK. ^9^Clinical Neuroscience Research Group, Greater Manchester Neurosciences Centre, University of Manchester, Salford, UK. ^10^Oxford Project to Investigate Memory and Ageing (OPTIMA), University of Oxford, Department of Pharmacology, Mansfield Road, Oxford OX3 9DU, UK. ^11^University of Bristol Institute of Clinical Neurosciences, School of Clinical Sciences, Frenchay Hospital, Bristol, UK. ^12^Department of Molecular Neuroscience and Reta Lilla Weston Laboratories, Institute of Neurology, London, UK. ^13^MRC Prion Unit, Department of Neurodegenerative Disease, UCL Institute of Neurology, London, UK. ^14^Dementia Research Centre, Department of Neurodegenerative Diseases, University College London, Institute of Neurology, London, UK.^15^Department of Psychiatry, University of Bonn, Sigmund-Freud-Straβe 25, 53105 Bonn, Germany. ^16^Institute of Primary Medical Care, University Medical Center Hamburg-Eppendorf, Germany. ^17^Department of Psychiatry, Charité Berlin, Germany. ^18^Department of Psychiatry, Friedrich-Alexander-University Erlangen-Nürnberg, Germany. ^19^Department of Psychiatry and Psychotherapy, University Medical Center (UMG), Georg-August-University, Göttingen, Germany. ^20^Institute for Stroke and Dementia Reserach, Klinikum der Universität München, Marchioninistr. 15, 81377, Munich, Germany. ^21^Department of Neurology, Klinikum der Universität München, Marchioninistr. 15, 81377, Munich, Germany. ^22^Central Institute of Mental Health, Medical Faculty Mannheim, University of Heidelberg, Germany. ^23^Discipline of Psychiatry, School of Medicine and Trinity College Institute of Neuroscience, Laboratory of Neuroimaging & Biomarker Research, Trinity College, University of Dublin, Ireland. ^24^Institute for Memory and Alzheimers Disease & INSERM, Sorbonne Universites, Pierre and Marie Curie University, Paris; Institute for Brain and Spinal Cord Disorders (ICM), Department of Neurology, Hospital of Pitié-Salpétrière. ^25^Centre for Geriatric Medicine and Section of Gerontopsychiatry and Neuropsychology, University of Freiburg, Germany. ^26^Department of Psychiatry, University of Halle, Halle, Germany. ^27^Departments of Psychiatry, Neurology and Genetics, Washington University School of Medicine, St Louis, MO 63110, US. ^28^Department of Biology, Brigham Young University, Provo, UT, 84602, USA. ^29^Neurodegenerative Brain Diseases group, Department of Molecular Genetics, VIB, Antwerpen, Belgium. ^30^Institute Born-Bunge and University of Antwerp; Antwerpen, Belgium. ^31^Memory Clinic and Department of Neurology, ZNA Middelheim, Antwerpen, Belgium. ^32^Department of Mental Health Sciences, University College London, UK. ^33^The Wellcome Trust Sanger Institute, Wellcome Trust Genome Campus, Hinxton, Cambridge, UK. ^34^MRC Centre for Neurodegeneration Research, Department of Clinical Neuroscience, Kings College London, Institute of Psychiatry, London, SE5 8AF, UK. ^35^Laboratory of Neurogenetics, National Institute on Aging, National Institutes of Health, Bethesda, MD, 20892, USA. ^36^Department of Genomics, Life & Brain Center, University of Bonn, Sigmund-Freud-Str. 25, D-53127 Bonn, Germany. ^37^Institute of Human Genetics, University of Bonn, Wilhelmstr. 31, D-53111 Bonn, Germany. ^38^Institute for Medical Informatics, Biometry and Epidemiology, University Hospital of Essen, University Duisburg-Essen, Hufelandstr. 55, D-45147 Essen, Germany. ^39^Institute of Epidemiology, Helmholtz Zentrum München, German Research Center for Environmental Health, 85764 Neuherberg, Germany. ^40^Institute of Medical Informatics, Biometry and Epidemiology, Chair of Epidemiology, Ludwig-Maximilians-Universität, Munich, Germany. ^41^Klinikum Grosshadern, Munich, Germany. ^42^Department of Neuroscience, Mayo Clinic College of Medicine, Jacksonville, Florida 32224, USA. ^43^Division of Biomedical Statistics and Informatics, Mayo Clinic and Mayo Foundation, Rochester, Minnesota 55905, USA. ^44^Department of Molecular Neuroscience, Institute of Neurology, University College London, Queen Square, London WC1N 3BG, UK. ^45^Deutsches Zentrum für Neurodegenerative Erkrankungen (DZNE), Bonn.

We also thank the EPIC InterAct collaborators: Claudia Langenberg(1), Robert A Scott(1), Stephen J Sharp(1), Nita G Forouhi(1), Nicola D Kerrison(1), Matt Sims(1), Debora ME Lucarelli(1), Inês Barroso(2,3), Panos Deloukas(4), Mark I McCarthy(5,6,7), Larraitz Arriola(8,9,10), Beverley Balkau(11,12), Aurelio Barricarte(13,10), Heiner Boeing(14), Paul W Franks(15,16), Carlos Gonzalez(17), Sara Grioni(18), Rudolf Kaaks(19), Timothy J Key(20), Carmen Navarro(21,10,22), Peter M Nilsson(15), Kim Overvad(23,24), Domenico Palli(25), Salvatore Panico(26), J. Ramón Quirós(27), Olov Rolandsson(28), Carlotta Sacerdote(29,30), Maria-José Sánchez(31,10,32), Nadia Slimani(33), Anne Tjonneland(34), Rosario Tumino(35,36), Daphne L van der A(37), Yvonne T van der Schouw(38), Elio Riboli(39), Nicholas J Wareham(1)

EPIC InterAct affiliations: (1) MRC Epidemiology Unit, University of Cambridge School of Clinical Medicine, Box 285 Institute of Metabolic Science, Cambridge Biomedical Campus Cambridge, CB2 0QQ, United Kingdom, (2) The Wellcome Trust Sanger Institute, Wellcome Trust Genome Campus, Hinxton, Cambridge, CB10 1SA, UK, (3) University of Cambridge Metabolic Research Laboratories, Institute of Metabolic Science Addenbrookeís Hospital CB2 0QQ Cambridge, (4) Department of Human Genetics, Wellcome Trust Sanger Institute, Wellcome Trust Genome Campus, Hinxton, Cambridge, CB10 1SA, United Kingdom, (5) Oxford Centre for Diabetes, Endocrinology and Metabolism (OCDEM), University of Oxford, Churchill Hospital, Old Road, Headington, Oxford, OX3 7LJ, UK, (6) Wellcome Trust Centre for Human Genetics, University of Oxford, Roosevelt Drive, Oxford, OX3 7BN, UK, (7) Oxford NIHR Biomedical Research Centre, Churchill Hospital, Old Road, Headington, Oxford, OX3 7LJ, UK, (8) Public Health Division of Gipuzkoa, Basque Government, Av. Navarra 4, 20013 San Sebastian, Spain, (9) Instituto BIO-Donostia, Basque Government, San Sebastian, Spain, (10) Consortium for Biomedical Research in Epidemiology and Public Health (CIBER EpidemiologÌa y Salud Pública), Melchor Fernández Almagro 3-5, 28029 Madrid, Spain, (11) Inserm, CESP Centre for research in Epidemiology and Population Health, U1018: Epidemiology of diabetes, obesity and chronic kidney disease over the lifecourse, 16 Av Paul Vaillant Couturier, 94807 Villejuif cedex, France , (12) Univ Paris Sud, UMRS 1018, F-94805, Villejuif, France, (13) Navarre Public Health Institute, Leyre 15, 31003 Pamplona, Navarra, Spain, (14) Department of Epidemiology, German Institute of Human Nutrition Potsdam-Rehbruecke, Arthur-Scheunert-Allee 114-116, 14558 Nuthetal, Germany, (15) Department of Clinical Sciences, Clinical Research Center, Skåne University Hospital, Lund University, 20502 Malmö, Sweden, (16) Department of Public Health and Clinical Medicine, UmeÂ University, 90187 Umeå, Sweden, (17) Unit Nutrition, Environment and Cancer, Department of Epidemiology, Catalan Institute of Oncology (ICO), Bellvitge Biomedical Research Institute (IDIBELL), Gran Via s/n 199-203, 08908 L'Hospitalet de Lolgbregat, Barcelona, Spain, (18) Fondazione IRCCS Istituto Nazionale dei Tumori Milan, Via Venezian, 1, 20133 Milan, Italy, (19) Division of Cancer Epidemiology, German Cancer Research Centre (DKFZ), Im Neuenheimer Feld 581, 69120 Heidelberg, Germany, (20) Cancer Epidemiology Unit, Nuffield Department of Population Health, University of Oxford, Oxford, United Kingdom, (21) Department of Epidemiology, Murcia Regional Health Council, Ronda de Levante, 11, 30008 Murcia, Spain, (22) Unit of Preventive Medicine and Public Health, School of Medicine, University of Murcia, Spain, (23) Department of Public Health, Section for Epidemiology, Aarhus University, Bartholins AllÈ 2, DK-8000 Aarhus C, Denmark, (24) Department of Cardiology, Aalborg University Hospital, Sdr. Skovvej 15, DK-9000 Aalborg, Denmark, (25) Molecular and Nutritional Epidemiology Unit, Cancer Research and Prevention Institute (ISPO), Via delle Oblate n.4 Padiglione 28A, 50141, Florence, Italy, (26) Dipartimento di Medicina Clinica e Chirurgia, Federico II University, via Pansini 5-80131 Naples, Italy, (27) Consejería de Sanidad, Public Health Directorate, C/Ciriaco Miguel Vigil 9, 33006- Oviedo-Asturias, Spain, (28) Department of Public Health and Clinical Medicine, Family Medicine, Umeå University 90187 Umeå, Sweden, (29) Unit of Cancer Epidemiology, AO Citta' della Salute e della Scienza Hospital-University of Turin and Center for Cancer Prevention (CPO), Via Santena 7, 10126 Torino, Italy, (30) Human Genetics Foundation (HuGeF), Via Nizza 52, 10126 Torino, Italy, (31) Andalusian School of Public Health, Cuesta del Observatorio 4, Ap. Correos 2070, 18080 Granada, Spain, (32) Instituto de Investigación Biosanitaria de Granada (Granada.ibs), Granada (Spain), (33) International Agency for Research on Cancer, Dietary Exposure Assessment Group (DEX), 150 Cours Albert Thomas, 69372 Lyon Cedex 08, France, (34) Danish Cancer Society Research Center, Strandboulevarden 49, 2100 Copenhagen, Denmark, (35) Cancer Registry and Histopathology Unit, "Civile - M.P. Arezzo" Hospital, Azienda Sanitaria Provinciale No 7, Via Dante Nr. 109, 97100 Ragusa, Italy, (36) Associazone Iblea per la Ricerca Epidemiologica - Onlus, Piazza Ancione No 2, 97100, Ragusa (Italy), (37) National Institute for Public Health and the Environment (RIVM), PO Box 1, 3720 BA Bilthoven, The Netherlands, (38) Julius Center for Health Sciences and Primary Care, University Medical Center Utrecht, Stratenum 6.131, PO Box 85.500, 3508 GA Utrecht, the Netherlands, (39) School of Public Health, Imperial College London, Norfolk Place, London W2 1PG, UK

We thank all EPIC participants and staff for their contribution to the study. We thank the lab team at the MRC Epidemiology Unit for sample management. Data on glycaemic traits have been contributed by MAGIC investigators and have been downloaded from [www.magicinvestigators.org](https://post.rm.dk/owa/redir.aspx?C=ecd3a0ca047341f286ade094eb8c6575&URL=http%3a%2f%2fwww.magicinvestigators.org).

**References**

1. Lambert JC, Ibrahim-Verbaas CA, Harold D, Naj AC, Sims R, et al. (2013) Meta-analysis of 74,046 individuals identifies 11 new susceptibility loci for alzheimer's disease. Nat Genet 45: 1452-1458.

2. Naj AC, Jun G, Beecham GW, Wang LS, Vardarajan BN, et al. (2011) Common variants at MS4A4/MS4A6E, CD2AP, CD33 and EPHA1 are associated with late-onset alzheimer's disease. Nat Genet 43: 436-441.

3. O'Dushlaine CT, Dolan C, Weale ME, Stanton A, Croke DT, et al. (2008) An assessment of the irish population for large-scale genetic mapping studies involving epilepsy and other complex diseases. Eur J Hum Genet 16: 176-183.

4. Purcell S, Neale B, Todd-Brown K, Thomas L, Ferreira MA, et al. (2007) PLINK: A tool set for whole-genome association and population-based linkage analyses. Am J Hum Genet 81: 559-575.

5. InterAct Consortium, Langenberg C, Sharp S, Forouhi NG, Franks PW, et al. (2011) Design and cohort description of the InterAct project: An examination of the interaction of genetic and lifestyle factors on the incidence of type 2 diabetes in the EPIC study. Diabetologia 54: 2272-2282.
